# Synthesis and self-assembly of dendritic–linear block copolymers containing poly(mandelic acid) with discrete molecular weights and stereochemical structures†

**DOI:** 10.1039/d3ra07536b

**Published:** 2024-01-10

**Authors:** Seul Woo Lee, Kyoung Taek Kim

**Affiliations:** a Department of Chemistry, Seoul National University Seoul 08826 Korea ktkim72@snu.ac.kr

## Abstract

In this work, we present the synthesis of uniform PMAs, where the number of repeat units and their stereochemical arrangement are precisely defined. Utilizing an iterative convergent approach with orthogonally protected dimandelic acid building blocks, we achieved high molecular weight PMAs with the desired number of repeat units, extending up to 144 mandelic acids. Additionally, stereochemically defined poly(l-mandelic acid)s with up to 32 repeat units were successfully synthesized. These uniform PMAs were subsequently coupled with uniform branched poly(ethylene glycol) blocks to create uniform dendritic–linear block copolymers. The self-assembly of these block copolymers in solution was systematically investigated. In solution self-assembly, the synthesized block copolymers showed multiple phases from cylinder to inverse cubic as the molecular weight of PMA increased. In the case of solvent diffusion-evaporation-mediated self-assembly, the block copolymers underwent a phase transition as the rate of water addition decreased.

## Abstract 2

Synthesis of PMAs with precisely defined molecular weights and stereochemical structures.

## Graphical abstract

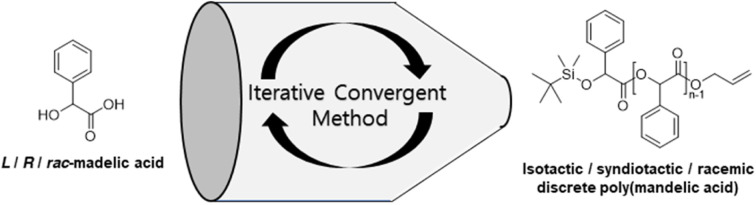

## Introduction

Poly(mandelic acid) (PMA) represents a biodegradable polymer that shares structural and property similarities with polystyrene (PS) but offers the additional benefit of environmental sustainability.^1–3^ Traditionally, PMA synthesis *via* bulk ring-opening polymerization of mandelide lacked control over its molecular weight and distribution. High-temperature ROP led to stereocenter epimerization and racemization.^4,5^ Fortunately, catalytic ROP of cyclic *O*-carboxyanhydrides (OCA) and 5-phenyl-1,3-dioxolane-4-one (Ph-DOX) has resolved these challenges, yielding well-defined PMAs in terms of molecular weight, stereoregularity, and chain topology.^5–10^ Thermal analyses of linear and cyclic PMAs have indicated high glass transition temperatures (*T*_g_ = 109 °C) and crystallinity (*T*_m_ = 180 °C) without compromising degradability.^11^ These results strongly support PMA as a biodegradable alternative to traditional plastics. However, recent syntheses of highly isotactic PMAs (*P*_m_ > 0.99) have resulted in varying melting temperatures (*T*_m_) and increased molecular weight (*M*_n_ > 10k), making it challenging to maintain low polydispersity (PDI > 1.2).^8–10^

**Fig. 1:**
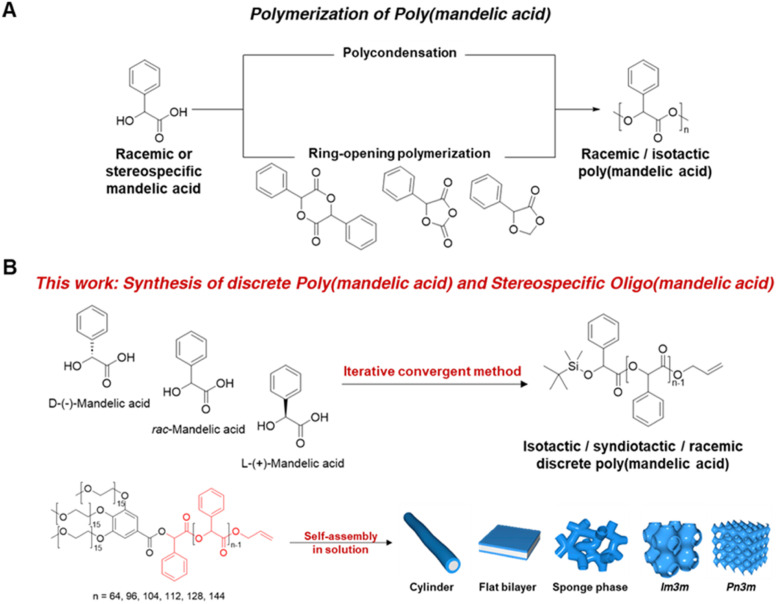
(A) Synthesis of racemic or isotactic poly(mandelic acid) *via* polycondensation and ring-opening polymerization (ref. 5–10). (B) Synthesis of racemic discrete PMA and isotactic/syndiotactic discrete oligo(mandelic acid) *via* iterative convergent method and solution self-assembly of dendritic–linear discrete 3mPEG15-*b*-uPMA.

In this study, we present the synthesis of PMAs with precisely defined molecular weight and stereochemical structures. This was achieved through an iterative exponential growth process utilizing orthogonally protected dimandelic acid as a building block. Our approach yielded uniform PMAs with up to 144 mandelic repeat units, preventing stereocenter epimerization. We also conducted thermal property and crystallization analyses of isotactic L-PMA. As an application example of PMAs as PS alternatives, we incorporated uniform PMAs with varying molecular weight poly(ethylene glycol) (PEG) segments into the PMA backbone and explored solution self-assembly (Fig. 1).

## Results & discussion

### Synthesis of discrete poly(mandelic acid)s

**Scheme 1:**
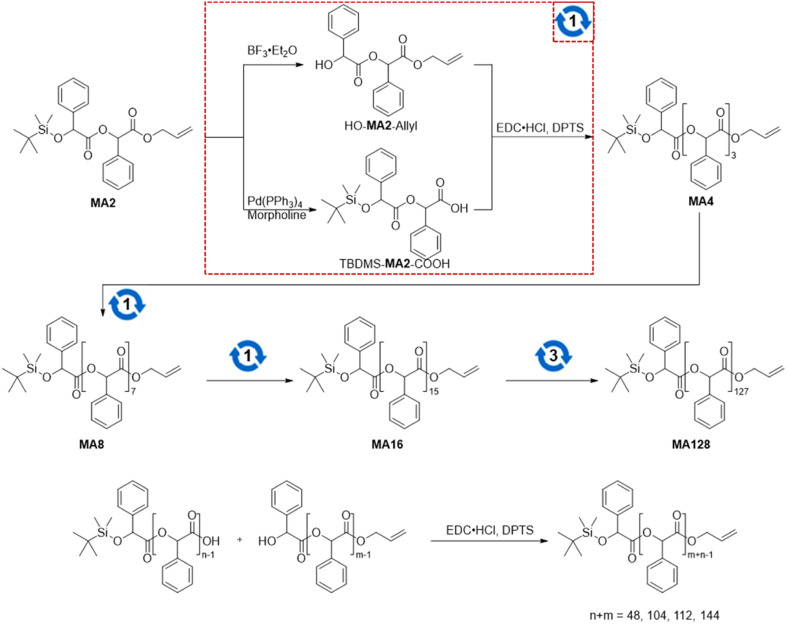
(upper) Iterative convergent synthesis of uPMA. The red dotted square indicates a convergence of linear precursors. The numbers inside circular arrows refer to the number of iterations of the convergence. (below) Hetero convergence of synthesizing uPMAs.

We synthesized poly(mandelic acid)s with uniform molecular weights (uPMAs) through the iterative convergent pathway, employing dimandelic acid with *t*-butylsilyl and allyl protecting groups as a building block (MA2 in Scheme 1). The allyl ester group was selected as a protecting group owing to susceptibility of mandelic acid to decomposition *via* hydrogenolysis. Subsequently, the allyl protecting group was removed through an allyl exchange reaction with piperidine in the presence of Pd(PPh_3_)_4_. Following orthogonal deprotection of MA2, the carbodiimide-mediated coupling of HO-MA2-allyl and TBDMS-MA2-COOH produced the tetramer of MA after purification (MA4 in Scheme 1). This iterative convergence and purification process led to the generation of oligomeric and polymeric MAs with discrete molecular weights (Scheme 1, upper). High molecular-weight uPMAs were purified using preparative size-exclusion chromatography (prep-SEC), capitalizing on the molecular weight differences between deprotected precursors and coupled products (Fig. S1 and S2†). Additionally, we synthesized uPMA with the desired molecular weight through cross-convergence between two precursors of different molecular weights (Scheme 1, below). All uPMAs underwent comprehensive characterization through ^1^H and ^13^C NMR, gel permeation chromatography (GPC), and matrix-assisted laser desorption ionization time-of-flight (MALDI-TOF) mass spectrometry, confirming their monodispersity in molecular weight (Fig. S5, S6, S12, S14–S23† and 2). Molecular weight values of all uPMAs analyzed by MALDI-TOF and GPC are summarized in Table 1. Poly(mandelic acid)s exhibit thermal characteristics similar to polystyrene, including glass transition temperature (*T*_g_).^1–5^ We determined the *T*_g_s of PMAs through differential scanning calorimetry (DSC). The measured *T*_g_s increased proportionally with the number of repeating units. *T*_g_s of MAn (*n* > 48) reached a plateau at 360 K (Fig. 3A). A Flory–Fox plot of the *T*_g_ of MA8–MA128 against the 1/*M* values (Fig. 3B) corroborated that the *T*_g_ of an infinitely long MAn (*T*_g,∞_ = 370.10 K) aligns with the literature values for amorphous PMAs.^5^

**Fig. 2:**
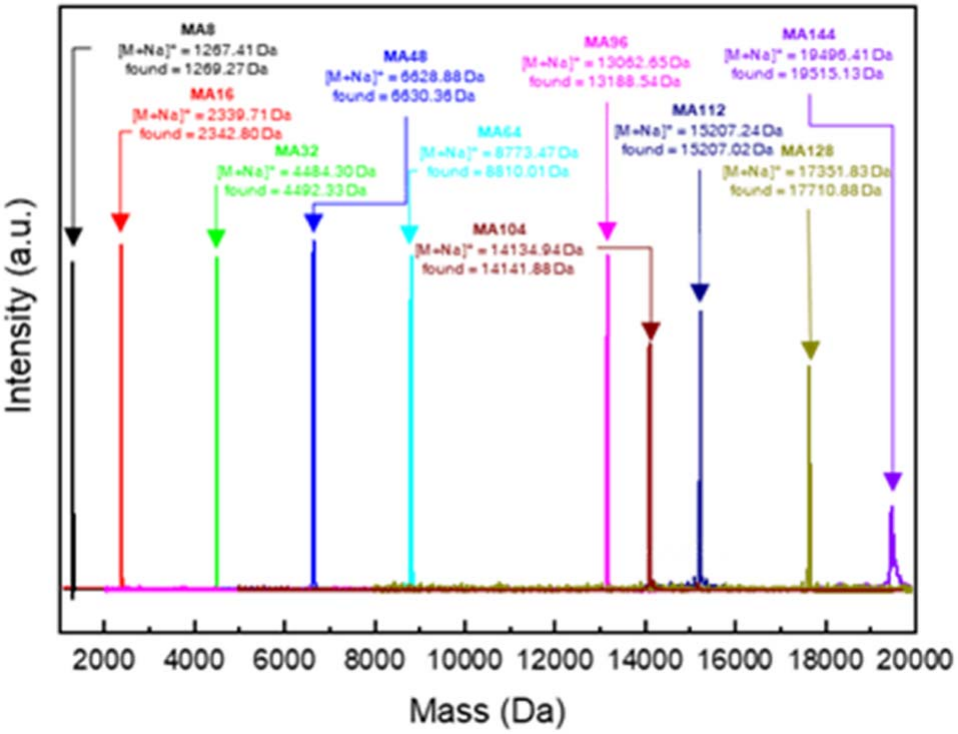
Combined MALDI-TOF mass spectra of uPMAs.

**Table 1:** Molecular weight analysis of uniform linear PMAs

Entry	MALDI-TOF		GPC		
	Calculated ([M + Na]^+^)	Found ([M + Na]^+^)	*M*_n_ a (kDa)	*M*_w_ a (kDa)	PDI^a^
MA16	2339.71	2342.80	2.10	2.14	1.02
MA32	4484.30	4487.45	3.95	4.07	1.03
MA48	6628.88	6630.36	6.06	6.30	1.04
MA64	8773.47	8810.01	7.64	8.17	1.07
MA96	13 062.65	13 188.54	11.70	12.17	1.04
MA104	14 134.94	14 141.88	12.91	13.56	1.05
MA112	15 207.24	15 207.02	13.74	14.43	1.05
MA128	17 351.83	17 710.88	15.35	16.12	1.05
MA144	19 496.41	19 515.13	15.84	17.27	1.09

a GPC was calibrated with a linear PS standard kit.

**Fig. 3:**
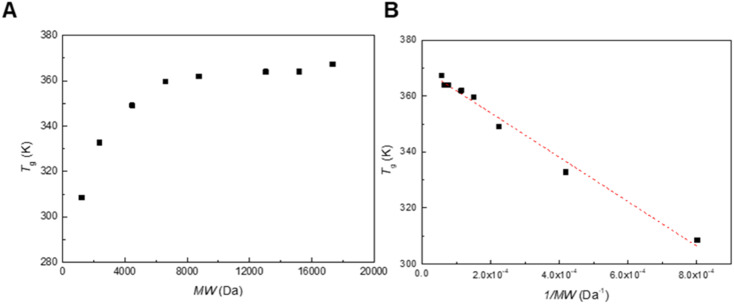
(A) *T*_g_*vs.* MW plots of linear uPMAs. (B) The Flory–Fox plots of linear uPMAs.

### Synthesis of isotactic and syndiotactic mandelic acid

Ring-opening polymerization of mandelide is prone to racemization of ⍺-hydrogen, resulting in stereoirregular PMAs.^12,13^ To overcome this issue, Buchard and coworkers reported that the ROP of cyclic *O*-carboxyanhydride (L-manOCA) could produce stereoregular PMAs.^6^ However, achieving absolute control of the stereochemistry of PMA at the monomer level through conventional ROPs remains challenging. Therefore, we synthesized isotactic and syndiotactic uPMA, with chains containing up to 32 l-mandelic acid (L-MA32).

**Scheme 2:**
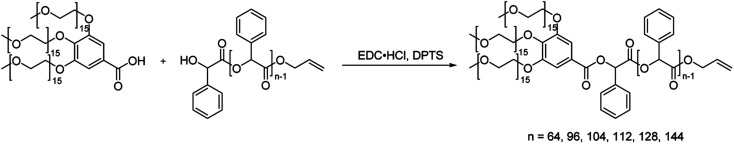
Synthesis of 3mPEG15-*b*-uPMA block copolymers.

**Fig. 4:**
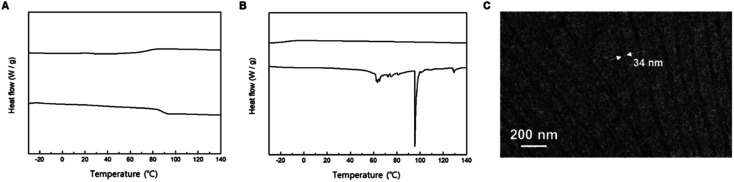
DSC (first heating and freezing scans, heat rate = 10 °C min^−1^) results of (A) L-MA32 and (B) 3mPEG-*b*-LMA32. (C) TEM image of 3mPEG15-*b*-LMA32 for crystallization-driven self-assembly in acetonitrile.

Employing iterative convergent pathways using isotactic and syndiotactic MA2 as building blocks, we successfully synthesized discrete oligo(mandelic acid)s with defined stereochemical arrangements (Scheme 1). The stereochemical configuration of the monomer units was determined through ^1^H and ^13^C NMR spectroscopy (Fig. S7 and S8†). DSC data unveiled distinct properties for isotactic and syndiotactic oligo(mandelic acid)s. Notably, L-MA8 exhibited a melting temperature (*T*_m_) at 57.9 °C, while DL-MA8 showed no *T*_m_ during the first heating and freezing scans (Fig. S35†). Based on these results, 3mPEG15-Me, L-MA32, and 3mPEG-*b*-LMA32 were synthesized (Scheme 1 and 2) and analyzed by DSC (Fig. 4A and B). L-MA32 showed *T*_g_ of 87.9 °C at first heating/freezing cycle,^15^ which is higher than that of amorphous MA32 (76.0 °C, Fig. 3A). Conversely, 3mPEG15-*b*-LMA32 showed a *T*_m_ of 95.4 °C at first heating cycle. Subsequently, crystallization-driven self-assembly (CDSA) of dendritic–linear block copolymers containing L-MA32 was performed.^14–17^ 3mPEG15-*b*-LMA32 was dissolved in acetonitrile (1 mg mL^−1^) and heated at 90 °C, followed by a slow cooling and equilibrium at 25 °C without perturbation for 24 h. The resulting solution was diluted by acetonitrile and analyzed through TEM. The diameters of more than 100 crystals were measured by analyzing the electron microscopy images.^18–20^ TEM images showed a crystalline structure with the diameter of *L*_n_ = 34.8 nm, *L*_w_ = 37.5 nm, and *L*_w_/*L*_n_ = 1.08 with various lengths (Fig. 4C and S36†).

### Synthesis of block copolymers and self-assembly

Solution self-assembly is a powerful method for exploiting the relationship between the chemical structure and self-assembly behavior of amphiphiles.^21–26^ In our previous studies, we investigated amphiphilic block copolymers comprising branched PEG and linear PS, where we observed inverse cubic phases with varying molecular weights.^27–30^ Based on the similarities in structures and properties between poly(mandelic acid) and polystyrene, we investigated the discrete molecular weight analogs of PEG-PS dendritic–linear BCPs using a branched PEG block and poly(mandelic acid)s without molecular weight distribution.

**Fig. 5:**
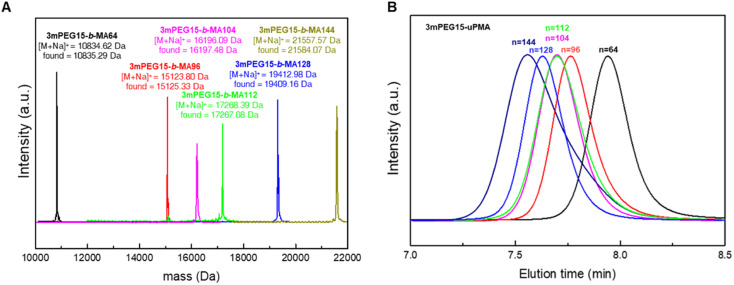
(A) Combined MALDI-TOF mass spectra of 3mPEG15-*b*-uPMAs. (B) GPC results for 3mPEG15-*b*-uPMAs indicating the absence of precursors left.

We began by synthesizing monomethoxy poly(ethylene glycol) with a uniform molecular weight distribution (mPEG), extending the chain length from 11 to 15 repeating units following the literature.^31^ By combining tosylated mPEG with 15 repeating units and methyl gallate, we successfully synthesized branched discrete poly(ethylene glycol) with 15 repeating units (3mPEG15). Characterization of the synthesized 3mPEG15 was conducted through ^1^H NMR, GPC, and MALDI-TOF (Fig. S9, S10, S13, and S24–S27†). 3mPEG15 served as the hydrophilic block for subsequent block copolymer synthesis. The carbodiimide-mediated coupling of 3mPEG15-COOH and HO-uPMA resulted in 3mPEG15-*b*-uPMA after purification (Scheme 2). All block copolymers underwent thorough characterization, including ^1^H and ^13^C NMR, GPC, and MALDI-TOF mass spectrometry, to ensure the absence of any remaining precursors (Fig. 5A, B and S28–S33†). Molecular weight values of all 3mPEG15-*b*-uPMAs analyzed by MALDI-TOF and GPC are summarized in Table 2.

**Table 2:** Molecular weight analysis of 3mPEG15-*b*-uPMAs

Entry	MALDI-TOF		GPC		
	Calculated ([M + Na]^+^)	Found ([M + Na]^+^)	*M*_n_ a (kDa)	*M*_w_ a (kDa)	PDI^a^
3mPEG15-*b*-MA64	10 834.62	10 835.29	10.54	10.86	1.03
3mPEG15-*b*-MA96	15 123.80	15 125.33	14.01	14.43	1.03
3mPEG15-*b*-MA104	16 196.09	16 197.48	15.38	16.00	1.04
3mPEG15-*b*-MA112	17 268.39	17 267.08	15.53	16.15	1.04
3mPEG15-*b*-MA128	19 412.98	19 409.16	17.58	18.11	1.03
3mPEG15-*b*-MA144	21 557.57	21 584.07	18.04	19.30	1.07
3mPEG15-*b*-LMA32	6545.45	6526.73	7.15	7.36	1.03

a GPC was calibrated with a linear PS standard kit.

**Fig. 6:**
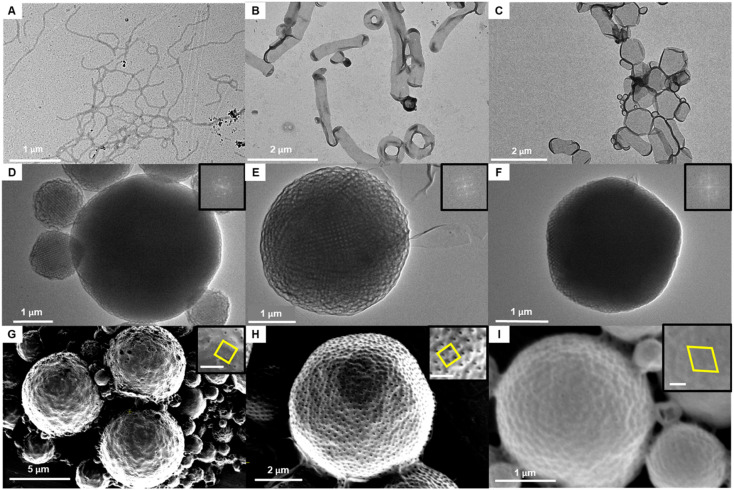
Phase transition of self-assembled structures of 3mPEG15-*b*-uPMA during solution self-assembly with increasing molecular weight of uPMA block. TEM images of (A) cylindrical micelle of 3mPEG15-*b*-MA64. (B) Flat bilayer and toroid of 3mPEG15-*b*-MA96. (C) Flat bilayer of 3mPEG15-*b*-MA104. (D) Polymeric cubosomes of 3mPEG15-*b*-MA112. (E) Polymeric cubosomes of 3mPEG15-*b*-MA128. (F) Polymeric cubosomes of 3mPEG15-*b*-MA144. The inset shows the fast Fourier transform (FFT) images. SEM images of (G) polymeric cubosomes of 3mPEG15-*b*-MA112 in microscopic scale. (H) Polymeric cubosomes of 3mPEG15-*b*-MA128 in microscopic scale. (I) Polymeric cubosomes of 3mPEG15-*b*-MA144 in microscopic scale. The insets show each patterns of pores on the surfaces. Scale bar is 200 nm.

Dendritic–linear block copolymers with uniform molecular weight, incorporating 3mPEG15 and uPMAs, were employed for solution self-assembly. Water was introduced (1 mL h^−1^) directly into the organic solvent containing the block copolymer (5 mg mL^−1^ in acetone), and phase transitions were monitored after dialysis in pure water for 24 h. As the molecular weight of uPMA increased, the self-assembled structures, analyzed by TEM and SEM, displayed a transition from cylindrical micelles to inverse cubic phases (Fig. 6). Compared to the use of dendritic–linear block copolymers containing polydisperse PEG and PS, low molecular weight uPMA exhibited an inverse cubic phase due to its high density (*ρ*_PMA_ = 1.25 *vs. ρ*_PS_ = 1.05 g cm^−3^)^5^ and high molecular weight of monomer, resulting in high packing parameter with similar molecular weights. In the range of 104 to 128 repeating units, a difference of 8 to 16 repeating units induced a phase transition in solution self-assembly. In the case of solution self-assembly of 3mPEG15-*b*-MA112 and 3mPEG15-*b*-MA128, *Im*3*m* phases were observed (Fig. 6D–E, G and H). In the case of 3mPEG15-*b*-MA144, phase transition from *Im*3*m* to *Pn*3*m* was observed, and the number of pores on the surface of particles reduced (Fig. 6F and I). In the range of 112 to 128 repeating units with acetone and 20% dioxane condition, *Im*3*m* phases were observed with particle and chunk formations in TEM and SEM images (Fig. S37 and S38†).

**Fig. 7:**
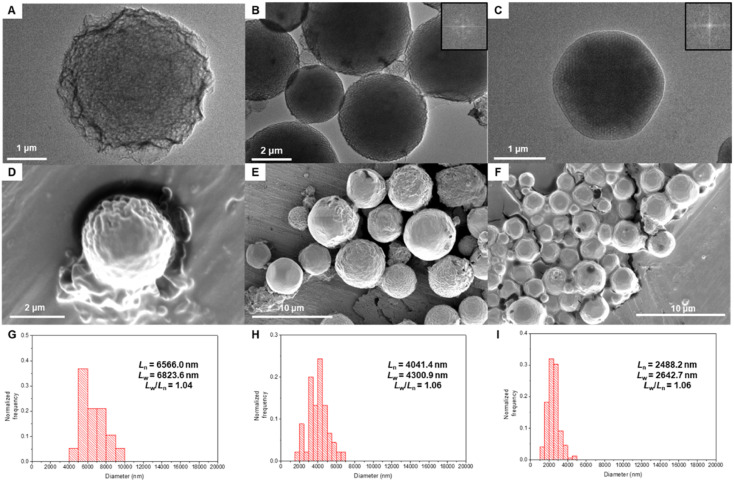
Phase transition of self-assembled structures of 3mPEG15-*b*-uPMA during solvent diffusion-evaporation self-assembly (SDESA) with increasing molecular weight of the uPMA block. TEM images of (A) sponge phase of 3mPEG15-*b*-MA96. (B) Polymeric cubosomes of 3mPEG15-*b*-MA112. (C) Polymeric cubosomes of 3mPEG15-*b*-MA128. The inset shows the fast Fourier transform (FFT) images. SEM images of (D) sponge phase of 3mPEG15-*b*-MA96 in the microscopic scale. (E) Polymeric cubosomes of 3mPEG15-*b*-MA112 in the microscopic scale. (F) Polymeric cubosomes with angular shapes of 3mPEG15-*b*-MA128 in microscopic scale. Histograms of diameter distribution in SDESA. (G) 3mPEG15-*b*-MA96; average diameter is 6566.0 nm, and PDI is 1.04. (H) 3mPEG15-*b*-MA112; average diameter is 4041.4 nm and PDI is 1.06. (I) 3mPEG15-*b*-MA128; average diameter is 2488.2 nm and PDI is 1.06.

To reduce the rate of water addition to the polymer solution,^29^ we conducted solvent diffusion-evaporation-mediated self-assembly (SDESA) for 3mPEG15-*b*-MA96, 3mPEG15-*b*-MA112, and 3mPEG15-*b*-MA128, using dioxane as the organic solvent. The organic solvent containing the block copolymer (0.5 mg mL^−1^) was placed in a small aluminum basket and located in a vial saturated with water. After 24 h, the organic solution became cloudy. Phase transitions were monitored after dialysis in pure water for 24 h. Comparatively, SDESA of 3mPEG15-*b*-MA96 exhibited a phase transition from flat bilayers to a sponge phase, while 3mPEG15-*b*-MA112 maintained *Im*3*m* symmetry (Fig. 7A and B). In contrast, 3mPEG15-*b*-MA128 underwent a transition from *Im*3*m* to *Pn*3*m* phases with angular shapes (Fig. 7C). Calculated from diameter measurements, the polydispersity index (PDI) of each block copolymer in SDESA displayed narrow values (PDI < 1.10, Fig. 7G–I and Table S8†).

## Conclusion

In summary, we successfully synthesized discrete poly(mandelic acid) with up to 144 repeating units and stereospecific discrete oligo(mandelic acid), including 32 repeating units of discrete poly(l-mandelic acid), using an iterative convergent method. Our research demonstrates that the iterative convergent method offers a means to synthesize poly(mandelic acid) with minimal polydispersity and precise control over stereochemistry, leading to the synthesis of isotactic and syndiotactic oligo(mandelic acid)s. Additionally, we synthesized dendritic–linear discrete 3mPEG-*b*-uPMA block copolymers. Our findings reveal that an increase in the hydrophobic block chain length, achieved by increasing uPMA repeating units, results in self-assemblies with higher-curvature structures and smaller molecular weights compared to using PS. These results indicate that the morphologies of block copolymers can be finely controlled by adjusting block ratios with an exact number of repeating units, utilizing discrete polymers.

## Conflicts of interest

There are no conflicts to declare.

## Supplementary Material

RA-014-D3RA07536B-s001
