# Supplementary material for: Synthesis and self-assembly of dendritic–linear block copolymers containing poly(mandelic acid) with discrete molecular weights and stereochemical structures
Source: RSC Adv. 2024 Jan 10;14(4):2285–92. doi: 10.1039/d3ra07536b (PMC10779440; doi:10.1039/d3ra07536b)
Supplement: RA-014-D3RA07536B-s001 [file RA-014-D3RA07536B-s001.pdf]

Electronic Supplementary Material (ESI) for RSC Advances.
This journal is © The Royal Society of Chemistry 2024

Supporting Information
for

Synthesis of Poly(mandelic acid) with Discrete Molecular Weights and their Solution Self-assembly with Dendritic-linear Block Copolymers

Seul Woo Lee[1,†] and Kyoung Taek Kim[1,*]

[1]Department of Chemistry, Seoul National University, Seoul 08826, Republic of Korea

*Correspondence to: ktkim72@snu.ac.kr

Contents

1. Method and Materials S-2

2. Iterative Convergent Synthesis of uPMAs and mPEG15-OH S-4

3. Synthesis of 3mPEG15-COOH and 3mPEG15-*b*-PMA S-16

4. Prep-SEC Separation of uPMAs and 3mPEG15-*b*-PMAs S-20

5. NMR Spectra of uPMAs, mPEGs, and their block copolymers S-24

6. GPC chromatograms of uPMAs and mPEGs S-29

7. MALDI-TOF Mass Spectra of uPMAs, mPEGs, and their block copolymers S-30

8. DSC results of Linear uPMAs S-40

9. Solution Self-assembly and crystallization-driven self-assembly of Block Copolymers S-41

10. References S-44

1. Method and Materials

Materials

rac-mandelic acid, benzyl bromide, allyl bromide, dimethylaminopyridine (DMAP), *p*-toluenesulfonic acid monohydrate, tetraethylene glycol, *p*-toluenesulfonyl chloride, anhydrous DMF (Alfa Aesar), *tert*-butyldimethylchlorosilane (Thermo fisher), triphenylmethyl chloride, 18-crwon-6 (Acros), potassium carbonate, imidazole and sodium hydroxide (Samchun chemicals) were used without further purification. Palladium on activated charcoal (10%), tetrakis(triphenylphosphine)palladium(0), sodium hydride, triethylene glycol monomethyl ether and methyl 3,4,5-trihydroxybenzoate were purchased from Sigma Aldrich and used without further purification. 1-(3-dimethylaminopropyl)-3-ethylcarbodiimide hydrochloride (EDC•HCl) was purchased from BLD pharm and used without purification. L-mandelic acid, D-mandelic acid, boron trifluoride diethyl ether and morpholine were purchased from Tokyo Chemical Industry and used without purification. CH_2Cl_2 (MC) was dried (using CaH_2 under a N_2 atmosphere) and distilled. Tetrahydrofuran (THF) was refluxed with Na and benzophenone under a N_2 atmosphere and distilled before used.

Characterization

^1H NMR and ^{13}C NMR spectra were recorded by Varian 500 MHz and Agilent 400-MR DD2 Magnetic Resonance System using $CDCl_3$ as solvent.

Gel permeation chromatography (GPC) was performed on an Agilent 1260 Infinity equipped with a PL gel 5 μm mixed D column and differential refractive index detectors. THF was used as an eluent with a flow rate of 1.0 mL min^{-1} at 35 °C. A polystyrene standard kit (Agilent Technologies) was used for calibration.

Mass spectra of linear uPMA and dendritic block copolymers were measured on a Bruker Ultraflex III TOF-TOF mass spectrometer equipped with a Nd:YAG laser (355 nm). The instrument was operated in linear and refractive positive ion modes. External calibration was conducted using the following peptides and proteins as reference: Bradykinin fragment 1-7 (757.3997 Da), Angiotensin II (1046.5423 Da), P14R (1533.8582 Da), ACTH fragment 18-39 (2465.1989 Da), Insulin oxidized B chain (3494.6513 Da), Insulin (5735 Da), Cytochrome c (12362 Da), Apomyoglobin (16952 Da), Aldolase (39212 Da) and Albumin (66430 Da)). The ion is $[M+H]^+$ ion. *trans*-2-[3-(4-*tert*-Butylphenyl)-2-methyl-2-propenylidene]malononitrile (DCTB) was used as a matrix.

Differential Scanning Calorimetry (DSC) was performed on a TA Instruments Q10 from –40

°C to 200 °C with a scan rate of 10 °C min^{-1} under N_2 atmosphere for **MAn** samples. Calibration was performed by measuring the melting temperature of indium (T_m = 429.75 K).
Transmission electron microscopy (TEM) was performed on a JEOL JEM-2100 operating at 120 kV. Specimens were prepared by placing a drop of the solution on a carbon-coated Cu grid (200 mesh, EM Science). The grid was air-dried overnight.
Scanning electron microscopy (SEM) was performed on a Thermofisher Scientific Apreo 2 S Hivac operating at 10 kV. The suspension was cast and dried on a SEM holder and coated with Pt using a Hitachi E-1030 ion sputter.

2. Iterative Convergent Synthesis of uPMAs and mPEGs

<u>General procedure for the iterative convergent synthesis of uPMAs</u>

General procedure for deallyllation giving carboxylic acid end functional group

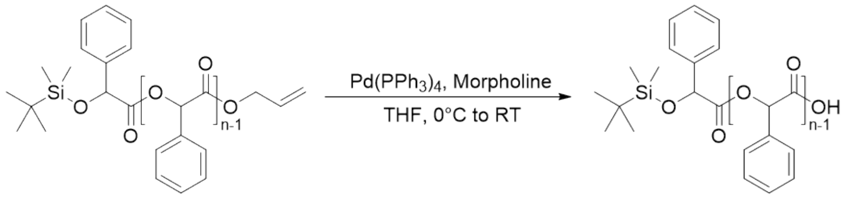

The allyl-protected compound was dissolved in dry THF (0.25 M) under argon. The solution was cooled to 0 ^{o}C in ice bath, tetrakis(triphenylphosphine)palladium(0) ($Pd(PPh_3)_4$, 0.05 eq.) and morpholine (1.05 eq.) were added, and the reaction mixture was stirred at room temperature. After completion, the solvent was removed under reduced pressure and dissolved in MC, and the mixture was washed with water, 1M HCl, and brine. The organic layer was separated, dried with $MgSO_4$, and filtered. The filtrate was concentrated under reduced pressure. After completion, diethyl ether was added and the palladium-suspended product was filtered through syringe filter. The solvent in the filtrate was removed under reduced pressure, giving deallyllated product.

General procedure for desilylation giving hydroxy end functional group

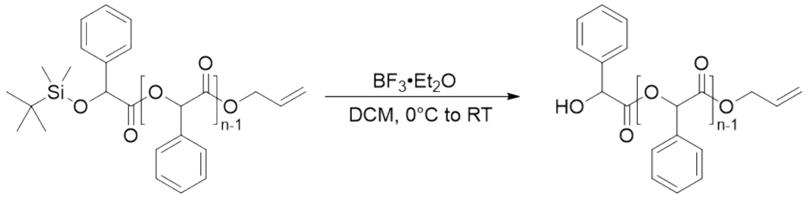

The TBDMS-protected compound was dissolved in dry DCM (0.5 M) under argon. The solution was cooled to 0 ^{o}C in ice bath, trifluoride diethyl etherate (BF_3•Et_2O, 5 eq.) was added slowly, and the reaction mixture was stirred at room temperature. After completion, saturated $NaHCO_3$ was poured into the mixture to quench the reaction, and the mixture was washed with brine. The organic layer was separated, dried with $MgSO_4$, and filtered. The filtrate was concentrated under reduced pressure. The crude product was purified by automated column chromatography using hexane/ethyl acetate as eluent giving the pure colorless product.

General procedure for esterification

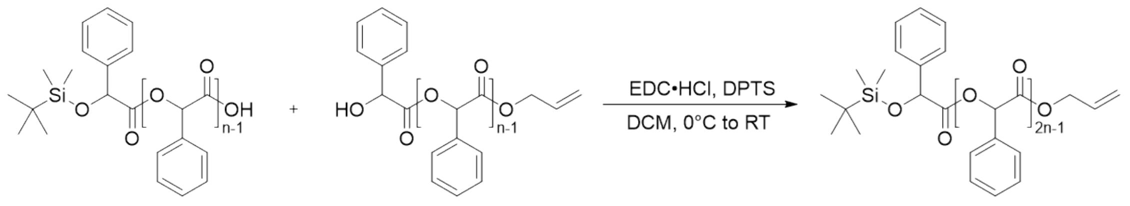

The hydroxy group and carboxylic acid group containing compounds were dissolved in dry DCM (0.5 M) under argon and cooled to 0 °C in ice bath. To the solution, 4-(dimethylamino)pyridinium 4-toluenesulfonate (DPTS, 0.3 eq.) and 1-(3-dimethylaminopropyl)-3-ethylcarbodiimide hydrochloride (EDC•HCl, 2.5 eq.) were added. The reaction mixture was stirred overnight at room temperature. After completion, the reaction mixture was washed with water and brine. The organic layer was dried with $MgSO_4$, filtered, and concentrated under reduced pressure. The crude product was purified by automated column chromatography using hexane/ethyl acetate ($n \leq 4$) and dichloromethane/ethyl acetate with 40% hexane ($n > 4$) as eluent. The purification of high molecular weight **MAn** ($n \geq 32$) was conducted by preparative size-exclusion chromatography (prep-SEC).

Synthesis of the constituting repeating unit **MA2**

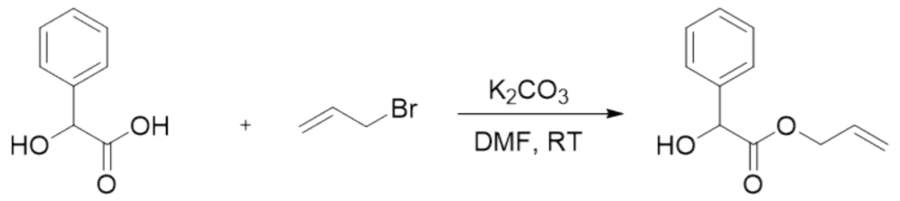

MA2 was synthesized as followed. HO-**MA**-Allyl: *Rac*-mandelic acid was dissolved in anhydrous DMF (1 M) under argon. To the solution, potassium carbonate (0.5 eq.) and allyl bromide (2 eq.) were added. The reaction mixture was stirred overnight at room temperature. After completion, water was added and the reaction mixture was washed with ethyl acetate. The organic layer was collected and washed with brine. The organic layer was dried with $MgSO_4$, filtered, and concentrated under reduced pressure. The crude product was purified by automated column chromatography using hexane/ethyl acetate as eluent. HO-**LMA**-Allyl and HO-**DMA**-Allyl follow the same procedure. Colorless liquid; ^1H NMR (500MHz, $CDCl_3$): δ 7.59-7.24 (m, 5H, Ph-**H**), 5.83 (m, 1H, COO-$CH_2C\mathbf{H}CH_2$), 5.18 (m, 3H, HO-C**H**(Ph)C=O and COO-$CH_2CHC\mathbf{H_2}$), 4.66 (m, 2H, COO-$C\mathbf{H_2}CHCH_2$), 3.55 (s, 1H, **H**O-CH(Ph)C=O) ppm; ^{13}C NMR (400MHz, $CDCl_3$): δ 173.2 (HO-CH(Ph)**C**=O), 138.4 ($\mathbf{C_6}H_5$-CH-COO), 131.3

(CH$_2$**C**HCH$_2$-O), 128.5 (**C$_6$H$_5$**-CH-COO), 128.4 (**C$_6$H$_5$**-CH-COO), 118.5 (**C**H$_2$CHCH$_2$-O), 73.0 (C$_6$H$_5$-**C**H-COO), 66.2 (CH$_2$CH**C**H$_2$-O) ppm.

HO-**LMA**-Allyl. White solid; ^{1}H NMR (500MHz, CDCl$_3$): δ 7.49-7.30 (m, 5H, Ph-**H**), 5.83 (ddt, 1H, COO-CH$_2$C**H**CH$_2$), 5.22-5.19 (m, 2H, COO-CH$_2$CHC**H$_2$**), 5.17 (dq, 1H, HO-C**H**(Ph)C=O), 4.66 (qdt, 2H, COO-C**H$_2$**CHCH$_2$), 3.45 (d, 1H, **H**O-CH(Ph)C=O) ppm; ^{13}C NMR (400MHz, CDCl$_3$): δ 173.5 (HO-CH(Ph)**C**=O), 138.4 (**C$_6$H$_5$**-CH-COO), 131.2 (CH$_2$**C**HCH$_2$-O), 128.8 (**C$_6$H$_5$**-CH-COO), 128.7 (**C$_6$H$_5$**-CH-COO), 126.7 (**C$_6$H$_5$**-CH-COO), 118.9 (**C**H$_2$CHCH$_2$-O), 73.0 (C$_6$H$_5$-**C**H-COO), 66.6 (CH$_2$CH**C**H$_2$-O) ppm.

HO-**DMA**-Allyl. White solid; ^{1}H NMR (500MHz, CDCl$_3$): δ 7.48-7.29 (m, 5H, Ph-**H**), 5.83 (m, 1H, COO-CH$_2$C**H**CH$_2$), 5.23-5.19 (m, 2H, COO-CH$_2$CHC**H$_2$**), 5.17 (d, 1H, HO-C**H**(Ph)C=O), 4.65 (qdt, 2H, COO-C**H$_2$**CHCH$_2$), 3.59 (d, 1H, **H**O-CH(Ph)C=O) ppm; ^{13}C NMR (400MHz, CDCl$_3$): δ 173.4 (HO-CH(Ph)**C**=O), 138.4 (**C$_6$H$_5$**-CH-COO), 131.2 (CH$_2$**C**HCH$_2$-O), 128.7 (**C$_6$H$_5$**-CH-COO), 128.6 (**C$_6$H$_5$**-CH-COO), 126.7 (**C$_6$H$_5$**-CH-COO), 118.8 (**C**H$_2$CHCH$_2$-O), 73.0 (C$_6$H$_5$-**C**H-COO), 66.6 (CH$_2$CH**C**H$_2$-O) ppm.

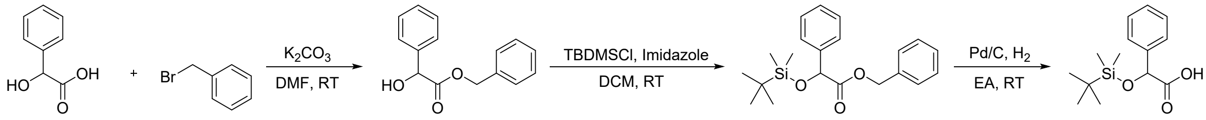

HO-**MA**-Bz: *Rac*-mandelic acid was dissolved in anhydrous DMF (1 M) under argon. To the solution, potassium carbonate (0.5 eq.) and benzyl bromide (2 eq.) were added. The reaction mixture was stirred overnight at room temperature. After completion, water was added and the reaction mixture was washed with ethyl acetate. The organic layer was collected and washed with brine. The organic layer was dried with MgSO$_4$, filtered, and concentrated under reduced pressure. The crude product was recrystallized in hexane/ethyl acetate cosolvent. HO-**LMA**-Bz and HO-**DMA**-Bz follow the same procedure. Colorless plate shaped crystal; ^{1}H NMR (500MHz, CDCl$_3$): δ 7.48-7.17 (m, 10H, Ph-**H**), 5.21 (m, 3H, HO-C**H**(Ph)C=O and Ph-C**H$_2$**-O), 3.57 (s, 1H, **H**O-CH(Ph)C=O) ppm; ^{13}C NMR (400MHz, CDCl$_3$): δ 173.6 (HO-CH(Ph)**C**=O), 138.3 (**C$_6$H$_5$**-CH-COO), 135.1 (**C$_6$**H$_5$-CH$_2$-O), 128.7 (**C$_6$H$_5$**-CH-COO), 128.4 (**C$_6$H$_5$**-CH$_2$-O), 73.1 (C$_6$H$_5$-**C**H-COO), 67.7 (C$_6$H$_5$-**C**H$_2$-O) ppm.

HO-**LMA**-Bz. Colorless plate shaped crystal; ^{1}H NMR (500MHz, CDCl$_3$): δ 7.47-7.18 (m, 10H, Ph-**H**), 5.21 (m, 3H, HO-C**H**(Ph)C=O and Ph-C**H$_2$**-O), 3.54 (s, 1H, **H**O-CH(Ph)C=O) ppm; ^{13}C NMR (400MHz, CDCl$_3$): δ 173.6 (HO-CH(Ph)**C**=O), 138.3 (**C$_6$H$_5$**-CH-COO), 135.1 (**C$_6$**H$_5$-CH$_2$-O), 128.7 (**C$_6$H$_5$**-CH-COO), 128.4 (**C$_6$H$_5$**-CH$_2$-O), 73.1 (C$_6$H$_5$-**C**H-COO), 67.7 (C$_6$H$_5$-**C**H$_2$-O) ppm.

HO-**DMA**-Bz. Colorless plate shaped crystal; ^{1}H NMR (500MHz, $CDCl_3$): δ 7.48-7.17 (m, 10H, Ph-**H**), 5.21 (m, 3H, HO-C**H**(Ph)C=O and Ph-C**H$_2$**-O), 3.57 (s, 1H, **H**O-CH(Ph)C=O) ppm; ^{13}C NMR (400MHz, $CDCl_3$): δ 173.6 (HO-CH(Ph)**C**=O), 138.3 (**C$_6$**H**$_5$**-CH-COO), 135.1 (**C$_6$**H_5-CH_2-O), 128.7 (**C$_6$**H**$_5$**-CH-COO), 128.4 (**C$_6$**H**$_5$**-CH_2-O), 73.1 (C_6H_5-**C**H-COO), 67.7 (C_6H_5-**C**H_2-O) ppm.

TBDMS-**MA**-Bz: HO-**MA**-Bz was dissolved in dry DCM (0.5 M) under argon. To the solution, imidzaole (1.5 eq.) and *tert*-butyldimethylchlorosilane (1.2eq.) were added. The reaction mixture was stirred overnight at room temperature. After completion, the reaction mixture was washed with water and brine. The organic layer was dried with $MgSO_4$, filtered, and concentrated under reduced pressure. The crude product was purified by automated column chromatography using hexane/ethyl acetate as eluent. TBDMS-**LMA**-Bz and TBDMS-**DMA**-Bz follow the same procedure. Colorless liquid; ^{1}H NMR (500MHz, $CDCl_3$): δ 7.60-7.17 (m, 10H, Ph-**H**), 5.33 (s, 1H, SiO-C**H**(Ph)C=O), 5.16 (s, 2h, Ph-C**H$_2$**-O), 0.96 (s, 9H, (C**H$_3$**)$_3$C-Si), 0.10 (d, 6H, $(CH_3)_3$C-Si(C**H$_3$**)$_2$-O) ppm; ^{13}C NMR (400MHz, $CDCl_3$): δ 171.9 (SiO-CH(Ph)**C**=O), 139.2 (**C$_6$**H**$_5$**-CH-COO), 135.8 (**C$_6$**H**$_5$**-CH-OSi) 128.5 (**C$_6$**H**$_5$**-CH-COO), 74.6 (SiO-**C**H(Ph)C=O), 66.7 (Ph-**C**H_2-O), 25.8 ($(CH_3)_3$**C**-Si$(CH_3)_2$-O), 18.4 ((**C**H$_3$)$_3$C-Si(CH**$_3$**)$_2$-O), -4.9 ($(CH_3)_3$C-Si(**C**H$_3$)$_2$-O), -5.3 ($(CH_3)_3$C-Si(**CH$_3$**)$_2$-O) ppm.

TBDMS-**LMA**-Bz. Colorless liquid; ^{1}H NMR (500MHz, $CDCl_3$): δ 7.56-7.20 (m, 10H, Ph-**H**), 5.30 (d, 1H, SiO-C**H**(Ph)C=O), 5.15 (d, 2h, Ph-C**H$_2$**-O), 0.93 (d, 9H, (C**H$_3$**)$_3$C-Si), 0.11 (d, 6H, $(CH_3)_3$C-Si(C**H$_3$**)$_2$-O) ppm; ^{13}C NMR (400MHz, $CDCl_3$): δ 172.1 (SiO-CH(Ph)**C**=O), 139.2 (**C$_6$**H**$_5$**-CH-COO), 135.8, 128.6, 128.5, 128.3, 128.1, 126.5 (**C$_6$**H**$_5$**-CH-OSi and **C$_6$**H**$_5$**-CH-COO), 74.6 (SiO-**C**H(Ph)C=O), 66.7 (Ph-**C**H_2-O), 25.8 ($(CH_3)_3$**C**-Si(CH**$_3$**)$_2$-O), 18.5 ((**C**H$_3$)$_3$C-Si(CH**$_3$**)$_2$-O), -4.9 ($(CH_3)_3$C-Si(**CH$_3$**)$_2$-O), -5.0 ($(CH_3)_3$C-Si(**CH$_3$**)$_2$-O) ppm.

TBDMS-**DMA**-Bz. Colorless liquid; ^{1}H NMR (500MHz, $CDCl_3$): δ 7.55-7.19 (m, 10H, Ph-**H**), 5.29 (d, 1H, SiO-C**H**(Ph)C=O), 5.13 (d, 2h, Ph-C**H$_2$**-O), 0.92 (d, 9H, (C**H$_3$**)$_3$C-Si), 0.10 (d, 6H, $(CH_3)_3$C-Si(C**H$_3$**)$_2$-O) ppm; ^{13}C NMR (400MHz, $CDCl_3$): δ 172.1 (SiO-CH(Ph)**C**=O), 139.2 (**C$_6$**H**$_5$**-CH-COO), 135.8, 128.6, 128.5, 128.3, 128.1, 126.5 (**C$_6$**H**$_5$**-CH-OSi and **C$_6$**H**$_5$**-CH-COO), 74.6 (SiO-**C**H(Ph)C=O), 66.8 (Ph-**C**H_2-O), 25.8 ($(CH_3)_3$**C**-Si(CH**$_3$**)$_2$-O), 18.5 ((**C**H$_3$)$_3$C-Si(CH**$_3$**)$_2$-O), -4.9 ($(CH_3)_3$C-Si(**CH$_3$**)$_2$-O), -5.0 ($(CH_3)_3$C-Si(**CH$_3$**)$_2$-O) ppm.

TBDMS-**MA**-COOH: TBDMS-**MA**-Bz was dissolved in ethyl acetate (0.5 M) and palladium on activated carbon (10 wt%, 0.05eq.) was added to the solution. After purged with nitrogen gas for a few minutes, hydrogen gas was purged into the reaction mixture using balloons and the reaction mixture was stirred at room temperature. After completion, the palladium-

suspended product was filtered through filter papers and syringe filter. The solvent in the filtrate was removed under reduced pressure. TBDMS-**LMA**-COOH and TBDMS-**DMA**-COOH follow the same procedure. Colorless liquid; ^{1}H NMR (500MHz, $CDCl_3$): δ 7.49-7.04 (m, 5H, Ph-**H**), 5.21 (s, 1H, SiO-C**H**(Ph)C=O), 0.89 (s, 9H, (C**H$_3$**)$_3$C-Si), 0.10 (d, 6H, $(CH_3)_3$C-Si(C**H$_3$**)$_2$-O) ppm; ^{13}C NMR (400MHz, $CDCl_3$): δ 171.2 (SiO-CH(Ph)**C**=O), 138.6 (**C$_6$H$_5$**-CH-COO), 135.8 (**C$_6$**H$_5$-CH-OSi) 128.6 (**C$_6$**H$_5$-CH-OSi), 74.3 (SiO-**C**H(Ph)C=O), 25.6 ((CH_3)$_3$**C**-Si(CH_3)$_2$-O), 18.2 ((**C**H$_3$)$_3$C-Si(CH_3)$_2$-O), -5.1 ((CH_3)$_3$C-Si(**C**H$_3$)$_2$-O), -5.3 ((CH_3)$_3$C-Si(**C**H$_3$)$_2$-O) ppm.

TDMBS-**LMA**-COOH. White solid; ^{1}H NMR (500MHz, $CDCl_3$): δ 7.50-7.30 (m, 5H, Ph-**H**), 5.23 (s, 1H, SiO-C**H**(Ph)C=O), 0.93 (s, 9H, (C**H$_3$**)$_3$C-Si), 0.12 (d, 6H, (CH_3)$_3$C-Si(C**H$_3$**)$_2$-O) ppm; ^{13}C NMR (400MHz, $CDCl_3$): δ 171.2 (SiO-CH(Ph)**C**=O), 138.2 (**C$_6$**H$_5$-CH-COO), 128.7, 126.6 (**C$_6$**H$_5$-CH-OSi), 74.4 (SiO-**C**H(Ph)C=O), 25.8 ((CH_3)$_3$**C**-Si(CH_3)$_2$-O), 18.3 ((**C**H$_3$)$_3$C-Si(CH_3)$_2$-O), -4.8 ((CH_3)$_3$C-Si(**C**H$_3$)$_2$-O), -5.1 ((CH_3)$_3$C-Si(**C**H$_3$)$_2$-O) ppm.

TDMBS-**DMA**-COOH. White solid; ^{1}H NMR (500MHz, $CDCl_3$): δ 7.50-7.31 (m, 5H, Ph-**H**), 5.21 (s, 1H, SiO-C**H**(Ph)C=O), 0.92 (s, 9H, (C**H$_3$**)$_3$C-Si), 0.12 (d, 6H, (CH_3)$_3$C-Si(C**H$_3$**)$_2$-O) ppm; ^{13}C NMR (400MHz, $CDCl_3$): δ 171.2 (SiO-CH(Ph)**C**=O), 138.1 (**C$_6$**H$_5$-CH-COO), 128.7, 126.5 (**C$_6$**H$_5$-CH-OSi), 74.5 (SiO-**C**H(Ph)C=O), 25.8 ((CH_3)$_3$**C**-Si(CH_3)$_2$-O), 18.3 ((**C**H$_3$)$_3$C-Si(CH_3)$_2$-O), -4.8 ((CH_3)$_3$C-Si(**C**H$_3$)$_2$-O), -5.1 ((CH_3)$_3$C-Si(**C**H$_3$)$_2$-O) ppm.

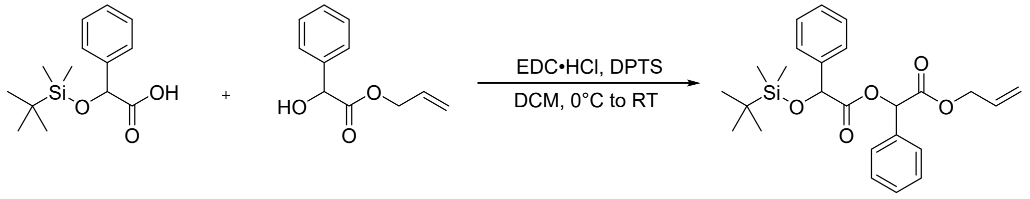

MA2: TBDMS-**MA**-COOH and HO-**MA**-Allyl were dissolved in dry DCM (0.5 M) under argon and cooled to 0 °C in ice bath. To the solution, 4-(dimethylamino)pyridinium 4-toluenesulfonate (DPTS, 0.3 eq.) and 1-(3-dimethylaminopropyl)-3-ethylcarbodiimide hydrochloride (EDC•HCl, 2.5 eq.) were added. The reaction mixture was stirred overnight at room temperature. After completion, the reaction mixture was washed with water and brine. The organic layer was dried with $MgSO_4$, filtered, and concentrated under reduced pressure. The crude product was purified by automated column chromatography using hexane/ethyl acetate as eluent. Colorless liquid; ^{1}H NMR (500MHz, $CDCl_3$): δ 7.64-7.21 (m, 10H, Ph-**H**), 5.96 (s, 1H, COO-C**H**(Ph)C=O), 5.76 (m, 1H, COO-CH_2C**H**CH_2), 5.42 (s, 1H, SiO-C**H**(Ph)C=O), 5.16 (m, 2H, COO-CH_2CHC**H$_2$**), 4.57 (m, 2H, CH_2CHC**H$_2$**-O), 0.95 (s, 9H,

(CH_3)$_3$C-Si), 0.12 (d, 6H, (CH_3)$_3$C-Si(CH_3)$_2$-O) ppm; ^{13}C NMR (400MHz, $CDCl_3$): δ 171.4 (SiO-CH(Ph)**C**=O), 168.1 (COO-CH(Ph)**C**=O), 138.7 (**C_6**H_5-CH-COO), 133.6 (**C_6**H_5-CH-COO), 131.3 (CH_2**C**HCH_2-O), 128.7 (**C_6**H_5-CH-COO), 118.5 (**C**H_2CHCH_2-O) 75.6 (C_6H_5-**C**H-SiO), 74.7 (C_6H_5-**C**H-COO), 66.0 (CH_2CH**C**H_2-O), 25.7 ((CH_3)$_3$**C**-Si(CH_3)$_2$-O), 18.3 ((**C**H_3)$_3$C-Si(CH_3)$_2$-O), -4.9 ((CH_3)$_3$C-Si(**C**H_3)$_2$-O), -5.2 ((CH_3)$_3$C-Si(**C**H_3)$_2$-O) ppm.

LMA2. Colorless liquid; ^{1}H NMR (500MHz, $CDCl_3$): δ 7.64-7.31 (m, 10H, Ph-**H**), 5.96 (s, 1H, COO-C**H**(Ph)C=O), 5.72 (ddt, 1H, COO-CH_2C**H**CH_2), 5.41 (s, 1H, SiO-C**H**(Ph)C=O), 5.22-5.09 (m, 2H, COO-CH_2CHC**H_2**), 4.53 (qdt, 2H, CH_2CHC**H_2**-O), 0.95 (s, 9H, (C**H_3**)$_3$C-Si), 0.12 (d, 6H, (CH_3)$_3$C-Si(C**H_3**)$_2$-O) ppm; ^{13}C NMR (400MHz, $CDCl_3$): δ 171.5 (SiO-CH(Ph)**C**=O), 167.9 (COO-CH(Ph)**C**=O), 138.7, 133.7 (**C_6**H_5-CH-COO), 131.4 (CH_2**C**HCH_2-O), 129.3, 128.8, 128.4, 128.3, 127.6, 126.7 (**C_6**H_5-CH-COO), 118.5 (**C**H_2CHCH_2-O) 75.1 (C_6H_5-**C**H-SiO), 74.3 (C_6H_5-**C**H-COO), 66.0 (CH_2CH**C**H_2-O), 25.8 ((CH_3)$_3$**C**-Si(CH_3)$_2$-O), 18.4 ((**C**H_3)$_3$C-Si(CH_3)$_2$-O), -4.9 ((CH_3)$_3$C-Si(**C**H_3)$_2$-O), -5.1 ((CH_3)$_3$C-Si(**C**H_3)$_2$-O) ppm.

DMA2. Colorless liquid; ^{1}H NMR (500MHz, $CDCl_3$): δ 7.64-7.30 (m, 10H, Ph-**H**), 5.96 (s, 1H, COO-C**H**(Ph)C=O), 5.72 (ddt, 1H, COO-CH_2C**H**CH_2), 5.42 (s, 1H, SiO-C**H**(Ph)C=O), 5.20-5.09 (m, 2H, COO-CH_2CHC**H_2**), 4.53 (qdt, 2H, CH_2CHC**H_2**-O), 0.96 (s, 9H, (C**H_3**)$_3$C-Si), 0.12 (d, 6H, (CH_3)$_3$C-Si(C**H_3**)$_2$-O) ppm; ^{13}C NMR (400MHz, $CDCl_3$): δ 171.5 (SiO-CH(Ph)**C**=O), 167.9 (COO-CH(Ph)**C**=O), 138.7, 133.7 (**C_6**H_5-CH-COO), 131.4 (CH_2**C**HCH_2-O), 129.3, 128.8, 128.4, 128.3, 127.6, 126.7 (**C_6**H_5-CH-COO), 118.5 (**C**H_2CHCH_2-O) 75.1 (C_6H_5-**C**H-SiO), 74.3 (C_6H_5-**C**H-COO), 66.0 (CH_2CH**C**H_2-O), 25.8 ((CH_3)$_3$**C**-Si(CH_3)$_2$-O), 18.4 ((**C**H_3)$_3$C-Si(CH_3)$_2$-O), -4.9 ((CH_3)$_3$C-Si(**C**H_3)$_2$-O), -5.1 ((CH_3)$_3$C-Si(**C**H_3)$_2$-O) ppm.

LDMA2. Colorless liquid; ^{1}H NMR (500MHz, $CDCl_3$): δ 7.60-7.24 (m, 10H, Ph-**H**), 5.96 (s, 1H, COO-C**H**(Ph)C=O), 5.79 (ddt, 1H, COO-CH_2C**H**CH_2), 5.43 (s, 1H, SiO-C**H**(Ph)C=O), 5.25-5.12 (m, 2H, COO-CH_2CHC**H_2**), 4.61 (qdt, 2H, CH_2CHC**H_2**-O), 0.94 (s, 9H, (C**H_3**)$_3$C-Si), 0.12 (d, 6H, (CH_3)$_3$C-Si(C**H_3**)$_2$-O) ppm; ^{13}C NMR (400MHz, $CDCl_3$): δ 171.4 (SiO-CH(Ph)**C**=O), 168.2 (COO-CH(Ph)**C**=O), 138.8, 133.6 (**C_6**H_5-CH-COO), 131.4 (CH_2**C**HCH_2-O), 129.2, 128.7, 128.4, 128.3, 127.4, 126.7 (**C_6**H_5-CH-COO), 118.5 (**C**H_2CHCH_2-O) 74.8 (C_6H_5-**C**H-SiO), 74.3 (C_6H_5-**C**H-COO), 66.1 (CH_2CH**C**H_2-O), 25.8 ((CH_3)$_3$**C**-Si(CH_3)$_2$-O), 18.4 ((**C**H_3)$_3$C-Si(CH_3)$_2$-O), -4.9 ((CH_3)$_3$C-Si(**C**H_3)$_2$-O), -5.1 ((CH_3)$_3$C-Si(**C**H_3)$_2$-O) ppm.

DLMA2. Colorless liquid; ^{1}H NMR (500MHz, $CDCl_3$): δ 7.57-7.27 (m, 10H, Ph-**H**), 5.94 (s, 1H, COO-C**H**(Ph)C=O), 5.78 (ddt, 1H, COO-CH_2C**H**CH_2), 5.42 (s, 1H, SiO-C**H**(Ph)C=O), 5.25-5.11 (m, 2H, COO-CH_2CHC**H_2**), 4.60 (qdt, 2H, CH_2CHC**H_2**-O), 0.93 (s, 9H, (C**H_3**)$_3$C-Si), 0.10 (d, 6H, (CH_3)$_3$C-Si(C**H_3**)$_2$-O) ppm; ^{13}C NMR (400MHz, $CDCl_3$): δ 171.4 (SiO-

CH(Ph)**C**=O), 168.1 (COO-CH(Ph)**C**=O), 138.7, 133.6 (**C_6H_5**-CH-COO), 131.3 (CH_2**C**HCH_2-O), 129.2, 128.7, 128.4, 128.3, 127.4, 126.7 (**C_6H_5**-CH-COO), 118.5 (**C**H_2CHCH_2-O) 74.8 (C_6H_5-**C**H-SiO), 74.3 (C_6H_5-**C**H-COO), 66.1 (CH_2CH**C**H_2-O), 25.8 ((CH_3)$_3$**C**-Si(CH_3)$_2$-O), 18.4 ((**C**H_3)$_3$C-Si(CH_3)$_2$-O), -4.9 ((CH_3)$_3$C-Si(**C**H_3)$_2$-O), -5.1 ((CH_3)$_3$C-Si(**C**H_3)$_2$-O) ppm.

Synthesis of uniform poly(mandelic acid) (uPMA)

TBDMS-**MAn**-COOH and HO-**MAn**-Allyl were dissolved in dry DCM (0.5 M) under argon and cooled to 0 °C in ice bath. To the solution, 4-(dimethylamino)pyridinium 4-toluenesulfonate (DPTS, 0.3 eq.) and 1-(3-dimethylaminopropyl)-3-ethylcarbodiimide hydrochloride (EDC•HCl, 2.5 eq.) were added. The reaction mixture was stirred overnight at room temperature. After completion, the reaction mixture was washed with water and brine. The organic layer was dried with $MgSO_4$, filtered, and concentrated under reduced pressure. The crude product was purified by automated column chromatography using hexane/ethyl acetate (n ≤ 4) and dichloromethane/ethyl acetate with 40% hexane (n > 4) as eluent. The purification of high molecular weight **MAn** (n ≥ 32) was conducted by preparative size-exclusion chromatography (prep-SEC). (TBDMS-**MAn**-COOH and HO-**MAn**-Allyl were prepared by the deallyllation and desilylation of **MAn** according to the described general procedure)

MA4. Colorless oil; ^{1}H NMR (500MHz, $CDCl_3$): δ 7.56-7.13 (m, 20H, Ph-**H**), 6.21-5.84 (m, 3H, COO-C**H**(Ph)C=O), 5.72 (m, 1H, COO-CH_2C**H**CH_2), 5.35 (s, 1H, SiO-C**H**(Ph)C=O), 5.13 (m, 2H, COO-CH_2CHC**H_2**), 4.53 (m, 2H, COO-C**H_2**CHCH_2), 0.89 (s, 9H, (C**H_3**)$_3$C-Si), 0.08 (m, 6H, (CH_3)$_3$C-Si(C**H_3**)$_2$-O) ppm; ^{13}C NMR (400MHz, $CDCl_3$): δ 170.9 (SiO-CH(Ph)**C**=O), 167.5 (COO-CH(Ph)**C**=O), 138.6 (**C_6H_5**-CH-COO), 133.3 (**C_6H_5**-CH-COO), 131.2 (COO-CH_2**C**HCH_2), 129.4-126.5 (**C_6H_5**-CH-COO), 118.6 (COO-CH_2CH**C**H_2) 75.3-74.3 (C_6H_5-**C**H-OSi and C_6H_5-**C**H-COO), 66.1 (COO-**C**H_2CHCH_2), 25.7 ((CH_3)$_3$**C**-Si(CH_3)$_2$-O), 18.3

((**C**H$_3$)$_3$C-Si(CH$_3$)$_2$-O), -5.1 ((CH$_3$)$_3$C-Si(**CH$_3$**)$_2$-O), -5.3 ((CH$_3$)$_3$C-Si(**CH$_3$**)$_2$-O) ppm. Yield: 23.71g, 75%

LMA4. Colorless oil; ^{1}H NMR (500MHz, CDCl$_3$): δ 7.63-7.29 (m, 20H, Ph-**H**), 6.11 (s, 2H, COO-C**H**(Ph)-COO-C**H**(Ph)-COO-CH$_2$CHCH$_2$), 5.92 (s, 1H, COO-C**H**(Ph)C=O), 5.74-5.61 (m, 1H, COO-CH$_2$C**H**CH$_2$), 5.39 (s, 1H, SiO-C**H**(Ph)C=O), 5.15-5.05 (m, 2H, COO-CH$_2$CHC**H$_2$**), 4.47 (qdt, 2H, COO-C**H$_2$**CHCH$_2$), 0.93 (s, 9H, (C**H$_3$**)$_3$C-Si), 0.08 (d, 6H, (CH$_3$)$_3$C-Si(C**H$_3$**)$_2$-O) ppm; ^{13}C NMR (400MHz, CDCl$_3$): δ 171.4 (SiO-CH(Ph)**C**=O), 167.5, 167.4, 167.1 (COO-CH(Ph)**C**=O), 138.6, 133.3, 132.7 (**C$_6$H$_5$**-CH-COO), 131.3 (COO-CH$_2$**C**HCH$_2$), 129.5-126.7 (**C$_6$H$_5$**-CH-COO), 118.5 (COO-CH$_2$CH**C**H$_2$) 75.3 (C$_6$H$_5$-**C**H-OSi), 74.9, 74.7, 74.3 (C$_6$H$_5$-**C**H-COO), 66.0 (COO-**C**H$_2$CHCH$_2$), 25.8 ((CH$_3$)$_3$**C**-Si(CH$_3$)$_2$-O), 18.4 ((**C**H$_3$)$_3$C-Si(CH$_3$)$_2$-O), -4.9 ((CH$_3$)$_3$C-Si(**CH$_3$**)$_2$-O), -5.1 ((CH$_3$)$_3$C-Si(**CH$_3$**)$_2$-O) ppm.

DMA4. Colorless oil; ^{1}H NMR (500MHz, CDCl$_3$): δ 7.59-7.23 (m, 20H, Ph-**H**), 6.09 (s, 2H, COO-C**H**(Ph)-COO-C**H**(Ph)-COO-CH$_2$CHCH$_2$), 5.89 (s, 1H, COO-C**H**(Ph)C=O), 5.71-5.58 (m, 1H, COO-CH$_2$C**H**CH$_2$), 5.37 (s, 1H, SiO-C**H**(Ph)C=O), 5.14-5.03 (m, 2H, COO-CH$_2$CHC**H$_2$**), 4.45 (qdt, 2H, COO-C**H$_2$**CHCH$_2$), 0.90 (s, 9H, (C**H$_3$**)$_3$C-Si), 0.10 (d, 6H, (CH$_3$)$_3$C-Si(C**H$_3$**)$_2$-O) ppm; ^{13}C NMR (400MHz, CDCl$_3$): δ 171.4 (SiO-CH(Ph)**C**=O), 167.5, 167.4, 167.2 (COO-CH(Ph)**C**=O), 138.6, 133.3, 132.7 (**C$_6$H$_5$**-CH-COO), 131.3 (COO-CH$_2$**C**HCH$_2$), 129.5-126.7 (**C$_6$H$_5$**-CH-COO), 118.5 (COO-CH$_2$CH**C**H$_2$) 75.3 (C$_6$H$_5$-**C**H-OSi), 74.9, 74.7, 74.3 (C$_6$H$_5$-**C**H-COO), 66.1 (COO-**C**H$_2$CHCH$_2$), 25.8 ((CH$_3$)$_3$**C**-Si(CH$_3$)$_2$-O), 18.4 ((**C**H$_3$)$_3$C-Si(CH$_3$)$_2$-O), -4.9 ((CH$_3$)$_3$C-Si(**CH$_3$**)$_2$-O), -5.1 ((CH$_3$)$_3$C-Si(**CH$_3$**)$_2$-O) ppm.

LDMA4. Colorless oil; ^{1}H NMR (500MHz, CDCl$_3$): δ 7.48-7.17 (m, 20H, Ph-**H**), 6.15 (d, 2H, COO-C**H**(Ph)-COO-C**H**(Ph)-COO-CH$_2$CHCH$_2$), 5.96 (s, 1H, COO-C**H**(Ph)C=O), 5.82-5.70 (m, 1H, COO-CH$_2$C**H**CH$_2$), 5.34 (s, 1H, SiO-C**H**(Ph)C=O), 5.22-5.11 (m, 2H, COO-CH$_2$CHC**H$_2$**), 4.58 (M, 2H, COO-C**H$_2$**CHCH$_2$), 0.89 (s, 9H, (C**H$_3$**)$_3$C-Si), 0.08 (d, 6H, (CH$_3$)$_3$C-Si(C**H$_3$**)$_2$-O) ppm; ^{13}C NMR (400MHz, CDCl$_3$): δ 171.0 (SiO-CH(Ph)**C**=O), 167.6, 167.4, 167.2 (COO-CH(Ph)**C**=O), 138.7, 133.3, 133.0 (**C$_6$H$_5$**-CH-COO), 131.3 (COO-CH$_2$**C**HCH$_2$), 129.4-126.8 (**C$_6$H$_5$**-CH-COO), 118.7 (COO-CH$_2$CH**C**H$_2$) 75.3 (C$_6$H$_5$-**C**H-OSi), 75.1, 74.5, 74.3 (C$_6$H$_5$-**C**H-COO), 66.2 (COO-**C**H$_2$CHCH$_2$), 25.8 ((CH$_3$)$_3$**C**-Si(CH$_3$)$_2$-O), 18.3 ((**C**H$_3$)$_3$C-Si(CH$_3$)$_2$-O), -4.9 ((CH$_3$)$_3$C-Si(**CH$_3$**)$_2$-O), -5.2 ((CH$_3$)$_3$C-Si(**CH$_3$**)$_2$-O) ppm.

DLMA4. Colorless oil; ^{1}H NMR (500MHz, CDCl$_3$): δ 7.50-7.16 (m, 20H, Ph-**H**), 6.15 (d, 2H, COO-C**H**(Ph)-COO-C**H**(Ph)-COO-CH$_2$CHCH$_2$), 5.96 (s, 1H, COO-C**H**(Ph)C=O), 5.86-

5.71 (m, 1H, COO-CH_2**C**HCH_2), 5.34 (s, 1H, SiO-C**H**(Ph)C=O), 5.25-5.06 (m, 2H, COO-CH_2CHC**H₂**), 4.58 (m, 2H, COO-C**H₂**CHCH_2), 0.89 (s, 9H, (C**H₃**)$_3$C-Si), 0.05 (d, 6H, $(CH_3)_3$C-Si(C**H₃**)$_2$-O) ppm; ^{13}C NMR (400MHz, $CDCl_3$): δ 171.0 (SiO-CH(Ph)**C**=O), 167.6, 167.4, 167.2 (COO-CH(Ph)**C**=O), 138.7, 133.2, 133.0 (**C_6H_5**-CH-COO), 131.3 (COO-CH_2**C**HCH_2), 129.4-126.8 (**C_6H_5**-CH-COO), 118.7 (COO-CH_2CH**C**H_2) 75.3 (C_6H_5-**C**H-OSi), 75.1, 74.5, 74.3 (C_6H_5-**C**H-COO), 66.2 (COO-**C**H_2CHCH_2), 25.8 ($(CH_3)_3$**C**-Si$(CH_3)_2$-O), 18.3 ((**C**H_3)$_3$C-Si$(CH_3)_2$-O), -4.9 ($(CH_3)_3$C-Si(**C**H_3)$_2$-O), -5.2 ($(CH_3)_3$C-Si(**C**H_3)$_2$-O) ppm.

MA8. Viscous oil; ^{1}H NMR (500MHz, $CDCl_3$): δ 7.68-7.10 (m, 40H, Ph-**H**), 6.30-5.94 (m, 7H, COO-C**H**(Ph)C=O), 5.76 (m, 1H, COO-CH_2C**H**CH_2), 5.47 (m, 1H, SiO-C**H**(Ph)C=O), 5.17 (m, 2H, COO-CH_2CHC**H₂**), 4.55 (m, 2H, COO-C**H₂**CHCH_2), 1.00 (s, 9H, (C**H₃**)$_3$C-Si), 0.15 (m, 6H, $(CH_3)_3$C-Si(C**H₃**)$_2$-O) ppm; MS (MALDI-TOF): m/z calcd for $C_{73}H_{68}O_{17}Si$ [**M**]: 1244.42; $C_{73}H_{68}O_{17}Si$+Na^+ [**M**+Na]$^+$: 1267.41; found 1269.27. Yield: 15.56g, 75%

LMA8. Colorless oil; ^{1}H NMR (500MHz, $CDCl_3$): δ 7.57-7.10 (m, 40H, Ph-**H**), 6.02 (m, 6H, COO-C**H**(Ph)C=O), 5.86 (s, 1H, COO-C**H**(Ph)C=O), 5.63 (m, 1H, COO-CH_2C**H**CH_2), 5.35 (s, 1H, SiO-C**H**(Ph)C=O), 5.07 (m, 2H, COO-CH_2CHC**H₂**), 4.43 (m, 2H, COO-C**H₂**CHCH_2), 0.89 (s, 9H, (C**H₃**)$_3$C-Si), 0.08 (d, 6H, $(CH_3)_3$C-Si(C**H₃**)$_2$-O) ppm

DMA8. Colorless oil; ^{1}H NMR (500MHz, $CDCl_3$): δ 7.55-7.08 (m, 40H, Ph-**H**), 6.05 (m, 6H, COO-C**H**(Ph)C=O), 5.87 (s, 1H, COO-C**H**(Ph)C=O), 5.64 (m, 1H, COO-CH_2C**H**CH_2), 5.36 (s, 1H, SiO-C**H**(Ph)C=O), 5.07 (m, 2H, COO-CH_2CHC**H₂**), 4.44 (m, 2H, COO-C**H₂**CHCH_2), 0.90 (s, 9H, (C**H₃**)$_3$C-Si), 0.09 (d, 6H, $(CH_3)_3$C-Si(C**H₃**)$_2$-O) ppm

LDMA8. Colorless oil; ^{1}H NMR (500MHz, $CDCl_3$): δ 7.47-7.00 (m, 40H, Ph-**H**), 6.10 (m, 6H, COO-C**H**(Ph)C=O), 5.95 (s, 1H, COO-C**H**(Ph)C=O), 5.74 (m, 1H, COO-CH_2C**H**CH_2), 5.32 (s, 1H, SiO-C**H**(Ph)C=O), 5.15 (m, 2H, COO-CH_2CHC**H₂**), 4.56 (m, 2H, COO-C**H₂**CHCH_2), 0.87 (s, 9H, (C**H₃**)$_3$C-Si), 0.08 (d, 6H, $(CH_3)_3$C-Si(C**H₃**)$_2$-O) ppm

DLMA8. Colorless oil; ^{1}H NMR (500MHz, $CDCl_3$): δ 7.47-7.03 (m, 40H, Ph-**H**), 6.10 (m, 6H, COO-C**H**(Ph)C=O), 5.95 (s, 1H, COO-C**H**(Ph)C=O), 5.75 (m, 1H, COO-CH_2C**H**CH_2), 5.31 (s, 1H, SiO-C**H**(Ph)C=O), 5.15 (m, 2H, COO-CH_2CHC**H₂**), 4.56 (m, 2H, COO-C**H₂**CHCH_2), 0.87 (s, 9H, (C**H₃**)$_3$C-Si), 0.08 (d, 6H, $(CH_3)_3$C-Si(C**H₃**)$_2$-O) ppm

MA16. Viscous oil; ^{1}H NMR (500MHz, $CDCl_3$): δ 7.59-6.96 (m, 80H, Ph-**H**), 6.18-5.82 (m, 15H, COO-C**H**(Ph)C=O), 5.73 (m, 1H, COO-CH_2C**H**CH_2), 5.34 (m, 1H, SiO-C**H**(Ph)C=O),

5.12 (m, 2H, COO-CH$_2$CHC**H$_2$**), 4.52 (m, 2H, COO-C**H$_2$**CHCH$_2$), 0.89 (s, 9H, (C**H$_3$**)$_3$C-Si), 0.08 (m, 6H, (CH$_3$)$_3$C-Si(C**H$_3$**)$_2$-O) ppm; M_n and $Đ$ (GPC): 2080 Da and 1.02; MS (MALDI-TOF): m/z calcd for $C_{137}H_{116}O_{33}Si$ [**M**]: 2316.72; $C_{137}H_{116}O_{33}Si+Na^+$ [**M**+Na]$^+$: 2339.71; found 2342.80. Yield: 12.60g, 87%

LMA16. Viscous oil; ^{1}H NMR (500MHz, CDCl$_3$): δ 7.55-7.01 (m, 80H, Ph-**H**), 6.09-5.94 (m, 14H, COO-C**H**(Ph)C=O), 5.86 (s, 1H, COO-C**H**(Ph)C=O), 5.63 (m, 1H, COO-CH$_2$C**H**CH$_2$), 5.32 (m, 1H, SiO-C**H**(Ph)C=O), 5.06 (m, 2H, COO-CH$_2$CHC**H$_2$**), 4.44 (m, 2H, COO-C**H$_2$**CHCH$_2$), 0.89 (s, 9H, (C**H$_3$**)$_3$C-Si), 0.08 (d, 6H, (CH$_3$)$_3$C-Si(C**H$_3$**)$_2$-O) ppm; MS (MALDI-TOF): m/z calcd for $C_{137}H_{116}O_{33}Si$ [**M**]: 2316.72; $C_{137}H_{116}O_{33}Si+Na^+$ [**M**+Na]$^+$: 2339.71; found 2340.70.

MA32. A white solid; ^{1}H NMR (500MHz, CDCl$_3$δ 7.58-6.92 (m, 160H, Ph-**H**), 6.06 (m, 31H, COO-C**H**(Ph)C=O), 5.71 (m, 1H, COO-CH$_2$C**H**CH$_2$), 5.33 (m, 1H, SiO-C**H**(Ph)C=O), 5.12 (m, 2H, COO-CH$_2$CHC**H$_2$**), 4.53 (m, 2H, COO-C**H$_2$**CHCH$_2$), 0.89 (s, 9H, (C**H$_3$**)$_3$C-Si), 0.08 (m, 6H, (CH$_3$)$_3$C-Si(C**H$_3$**)$_2$-O) ppm; M_n and $Đ$ (GPC): 3947 Da and 1.03; MS (MALDI-TOF): m/z calcd for $C_{265}H_{212}O_{65}Si$ [**M**]: 4461.31; $C_{265}H_{212}O_{65}Si+Na^+$ [**M**+Na]$^+$: 4484.30; found 4492.33. Yield: 8.49g, 70%

LMA32. A white solid; ^{1}H NMR (500MHz, CDCl$_3$δ 7.53-6.93 (m, 160H, Ph-**H**), 6.09-5.89 (m, 30H, COO-C**H**(Ph)C=O), 5.83 (s, 1H, COO-C**H**(Ph)C=O), 5.61 (m, 1H, COO-CH$_2$C**H**CH$_2$), 5.32 (m, 1H, SiO-C**H**(Ph)C=O), 5.06 (m, 2H, COO-CH$_2$CHC**H$_2$**), 4.41 (m, 2H, COO-C**H$_2$**CHCH$_2$), 0.86 (s, 9H, (C**H$_3$**)$_3$C-Si), 0.05 (d, 6H, (CH$_3$)$_3$C-Si(C**H$_3$**)$_2$-O) ppm; MS (MALDI-TOF): m/z calcd for $C_{265}H_{212}O_{65}Si$ [**M**]: 4461.31; $C_{265}H_{212}O_{65}Si+Na^+$ [**M**+Na]$^+$: 4484.30; found 4486.18.

MA48. A white solid; ^{1}H NMR (500MHz, CDCl$_3$): δ 7.46-6.94 (m, 240H, Ph-**H**), 6.02 (m, 47H, COO-C**H**(Ph)C=O), 5.73 (m, 1H, COO-CH$_2$C**H**CH$_2$), 5.29 (m, 1H, SiO-C**H**(Ph)C=O), 5.10 (m, 2H, COO-CH$_2$CHC**H$_2$**), 4.49 (m, 2H, COO-C**H$_2$**CHCH$_2$), 0.86 (s, 9H, (C**H$_3$**)$_3$C-Si), -0.02 (m, 6H, (CH$_3$)$_3$C-Si(C**H$_3$**)$_2$-O) ppm; M_n and $Đ$ (GPC): 6064 Da and 1.04; MS (MALDI-TOF): m/z calcd for $C_{393}H_{308}O_{97}Si$ [**M**]: 6605.89; $C_{393}H_{308}O_{97}Si+Na^+$ [**M**+Na]$^+$: 6628.88; found 6630.36. Yield: 0.78g, 72%

MA64. A white solid; ^{1}H NMR (500MHz, CDCl$_3$): δ 7.53-6.95 (m, 320H, Ph-**H**), 6.02 (m, 63H, COO-C**H**(Ph)C=O), 5.73 (m, 1H, COO-CH$_2$C**H**CH$_2$), 5.29 (m, 1H, SiO-C**H**(Ph)C=O), 5.09 (m, 2H, COO-CH$_2$CHC**H$_2$**), 4.49 (m, 2H, COO-C**H$_2$**CHCH$_2$), 0.88 (s, 9H, (C**H$_3$**)$_3$C-Si), -

0.07 (m, 6H, $(CH_3)_3C$-Si(C**H$_3$**)$_2$-O) ppm; M_n and $Đ$ (GPC): 7643 Da and 1.07; MS (MALDI-TOF): m/z calcd for $C_{521}H_{404}O_{129}Si$ [**M**]: 8750.48; $C_{521}H_{404}O_{129}Si+Na^+$ [**M**+Na]$^+$: 8773.47; found 8799.84. Yield: 5.82g, 70%

MA96. A white solid; ^{1}H NMR (500MHz, $CDCl_3$): δ 7.41-6.95 (m, 480H, Ph-**H**), 6.02 (m, 95H, COO-C**H**(Ph)C=O), 5.73 (m, 1H, COO-CH_2C**H**CH_2), 5.30 (m, 1H, SiO-C**H**(Ph)C=O), 5.09 (m, 2H, COO-CH_2CHC**H$_2$**), 4.50 (m, 2H, COO-C**H$_2$**CHCH_2), 0.86 (s, 9H, (C**H$_3$**)$_3$C-Si), -0.07 (m, 6H, $(CH_3)_3C$-Si(C**H$_3$**)$_2$-O) ppm; M_n and $Đ$ (GPC): 11700 Da and 1.04; MS (MALDI-TOF): m/z calcd for $C_{777}H_{596}O_{193}Si$ [**M**]: 13039.66; $C_{777}H_{596}O_{193}Si+Na^+$ [**M**+Na]$^+$: 13062.65; found 13188.54. Yield: 0.31g, 65%

MA104. A white solid; ^{1}H NMR (500MHz, $CDCl_3$): δ 7.44-6.94 (m, 520H, Ph-**H**), 6.01 (m, 103H, COO-C**H**(Ph)C=O), 5.73 (m, 1H, COO-CH_2C**H**CH_2), 5.30 (m, 1H, SiO-C**H**(Ph)C=O), 5.09 (m, 2H, COO-CH_2CHC**H$_2$**), 4.50 (m, 2H, COO-C**H$_2$**CHCH_2), 0.86 (s, 9H, (C**H$_3$**)$_3$C-Si), 0.01 (m, 6H, $(CH_3)_3C$-Si(C**H$_3$**)$_2$-O) ppm; M_n and $Đ$ (GPC): 12908 Da and 1.05; MS (MALDI-TOF): molecular m/z calcd for $C_{841}H_{644}O_{209}Si$ [**M**]: 14111.95; $C_{841}H_{644}O_{209}Si+Na^+$ [**M**+Na]$^+$: 14134.94; found 14141.88. Yield: 0.10g, 70%

MA112. A white solid; ^{1}H NMR (500MHz, $CDCl_3$): δ 7.44-6.94 (m, 560H, Ph-**H**), 6.01 (m, 111H, COO-C**H**(Ph)C=O), 5.73 (m, 1H, COO-CH_2C**H**CH_2), 5.30 (m, 1H, SiO-C**H**(Ph)C=O), 5.09 (m, 2H, COO-CH_2CHC**H$_2$**), 4.50 (m, 2H, COO-C**H$_2$**CHCH_2), 0.86 (s, 9H, (C**H$_3$**)$_3$C-Si), 0.01 (m, 6H, $(CH_3)_3C$-Si(C**H$_3$**)$_2$-O) ppm; M_n and $Đ$ (GPC): 13744 Da and 1.05; MS (MALDI-TOF): molecular m/z calcd for $C_{905}H_{692}O_{225}Si$ [**M**]: 15184.25; $C_{905}H_{692}O_{225}Si+Na^+$ [**M**+Na]$^+$: 15207.24; found 15207.02. Yield: 0.16g, 62%

MA128. A white solid; ^{1}H NMR (500MHz, $CDCl_3$): δ 7.46-6.85 (m, 640H, Ph-**H**), 6.00 (m, 127H, COO-C**H**(Ph)C=O), 5.73 (m, 1H, COO-CH_2C**H**CH_2), 5.30 (m, 1H, SiO-C**H**(Ph)C=O), 5.15 (m, 2H, COO-CH_2CHC**H$_2$**), 4.50 (m, 2H, COO-C**H$_2$**CHCH_2), 0.87 (s, 9H, (C**H$_3$**)$_3$C-Si), 0.01 (m, 6H, $(CH_3)_3C$-Si(C**H$_3$**)$_2$-O) ppm; M_n and $Đ$ (GPC): 15351 Da and 1.05; MS (MALDI-TOF): molecular m/z calcd for $C_{1033}H_{788}O_{257}Si$ [**M**]: 17328.84; $C_{1033}H_{788}O_{257}Si+Na^+$ [**M**+Na]$^+$: 17351.83; found 17710.88. Yield: 0.34g, 45%

MA144. A white solid; ^{1}H NMR (500MHz, $CDCl_3$): δ 7.44-6.89 (m, 720H, Ph-**H**), 5.98 (m, 143H, COO-C**H**(Ph)C=O), 5.73 (m, 1H, COO-CH_2C**H**CH_2), 5.30 (m, 1H, SiO-C**H**(Ph)C=O), 5.15 (m, 2H, COO-CH_2CHC**H$_2$**), 4.50 (m, 2H, COO-C**H$_2$**CHCH_2), 0.86 (s, 9H, (C**H$_3$**)$_3$C-Si), 0.01 (m, 6H, $(CH_3)_3C$-Si(C**H$_3$**)$_2$-O) ppm; M_n and $Đ$ (GPC): 15844 Da and 1.09; MS (MALDI-

TOF): molecular m/z calcd for $C_{1161}H_{884}O_{289}Si$ [**M**]: 19473.42; $C_{1161}H_{884}O_{289}Si+Na^+$ [**M**+Na]$^+$: 19496.41; found 19515.13. Yield: 0.2g, 50%

Table S1. Molecular weight analysis of uniform linear PMAs.

Entry	MALDI-TOF		GPC		
	calculated ([M+Na]$^+$)	found ([M+Na]$^+$)	M_n^{a} (kDa)	M_w^{a} (kDa)	PDI^a
**MA16**	2339.71	2342.80	2.10	2.14	1.02
**MA32**	4484.30	4487.45	3.95	4.07	1.03
**MA48**	6628.88	6630.36	6.06	6.30	1.04
**MA64**	8773.47	8810.01	7.64	8.17	1.07
**MA96**	13062.65	13188.54	11.70	12.17	1.04
**MA104**	14134.94	14141.88	12.91	13.56	1.05
**MA112**	15207.24	15207.02	13.74	14.43	1.05
**MA128**	17351.83	17710.88	15.35	16.12	1.05
**MA144**	19496.41	19515.13	15.84	17.27	1.09

[a] GPC was calibrated with a linear PS standard kit.

Synthesis of uniform poly(ethylene glycol) monomethyl ether (mPEG)

mPEG15-OH was synthesized according to the literature.[1] Starting from octa(ethylene glycol) trityl ether *p*-toluenesulfonate, addition of tetraethylene glycol was proceeded followed by tosylation, addition of triethylene glycol monomethyl ether, and deprotection of trityl group. Colorless oil; ^{1}H NMR (500MHz, $CDCl_3$): δ 3.62 (m, 60H, O-C**H$_2$**C**H$_2$**-O), 3.35 (s, 3H, O-CH_2CH_2-O-C**H$_3$**), 2.66 (s, 1H, **H**O-CH_2CH_2-O) ppm; ^{13}C NMR (400MHz, $CDCl_3$): δ 72.6 (CH_3-O-**C**H_2CH_2-O), 72.0 (CH_3-O-CH_2**C**H_2-O), 70.7 (O-**C**H_2**C**H_2-O), 61.8 (HO-**C**H_2CH_2-O), 59.1(**C**H_3-O-CH_2CH_2-O) ppm; M_n and $Đ$ (GPC): 857 Da and 1.02; MS (MALDI-TOF): molecular m/z calcd for $C_{31}H_{64}O_{16}$ [**M**]: 692.42; $C_{31}H_{64}O_{16}+Na^+$ [**M**+Na]$^+$: 715.41; found 713.34. Yield: 9.89g, 81%

3. Synthesis of 3mPEG15-COOH and 3mPEG15-*b*-MAn

General procedure fot synthesis of dendritic 3mPEG15-COOH

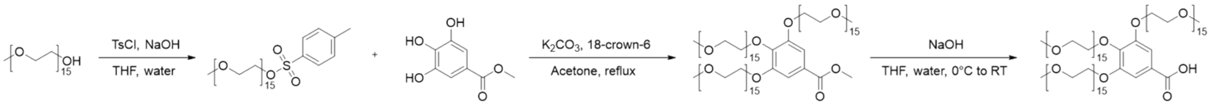

3mPEG15-COOH was synthesized as followed. **mPEG15-**Ts: **mPEG15**-OH was dissolved in THF (0.5 M) and cooled to 0 °C in ice bath. To the solution, sodium hydroxide solution (4 M, 4.5 eq.) was added slowly and stirred for 30 min. *p*-toluenesulfonyl chloride (2 eq.) was dissolved in THF and added to the solution. The reaction mixture was stirred overnight at room temperature. After completion, the reaction mixture was concentrated under reduced pressure and diluted with DCM, washed with water and brine. The organic layer was dried with Na_2SO_4, filtered, and concentrated under reduced pressure. The crude product was purified by automated column chromatography using ethyl acetate/methanol as eluent.

mPEG15-Ts. A colorless liquid; ^{1}H NMR (500MHz, $CDCl_3$): δ 7.77 (d, 2H, CH_3-C_6**H_4**-SO_3), 7.32 (d, 2H, CH_3-C_6**H_4**-SO_3), 4.13 (t, 2H, SO_3-C**H_2**CH_2-O), 3.83-3.40 (m, 58H, SO_3-CH_2C**H_2**-O and O-C**H_2**C**H_2**-O), 3.35 (d, 3H, O-C**H_3**), 2.42 (s, 3H, C**H_3**-C_6H_4-SO_3) ppm; ^{13}C NMR (400MHz, $CDCl_3$): δ 144.9 (CH_3-**C_6**H_4-SO_3), 133.1 (CH_3-**C_6**H_4-SO_3), 129.9 (CH_3-**C_6**H_4-SO_3), 128.1 (CH_3-**C_6**H_4-SO_3), 72.0 (CH_3-O-**C**$H_2$$CH_2$-O), 70.8-70.6 ($CH_3$-O-$CH_2$**C**$H_2$-O and O-**C**$H_2$**C**$H_2$-O), 69.3 ($SO_3$-$CH_2$**C**$H_2$-O), 68.7 ($SO_3$-$CH_2$**C**$H_2$-O), 59.1 (**C**$H_3$-O-$CH_2CH_2$-O), 21.7 (**C**$H_3$-$C_6H_4$-$SO_3$) ppm M_n and $Đ$ (GPC): 1008 Da and 1.02; MS (MALDI-TOF): molecular m/z calcd for $C_{38}H_{70}O_{18}S$ [**M**]: 846.43; $C_{38}H_{70}O_{18}S+Na^+$ [**M**+Na]$^+$: 869.42; found 867.23. Yield: 8.95g, 74%

3mPEG15-Me: **mPEG15**-Ts was dissovled in acetone (0.1 M) and 3,4,5-trihydroxybenzoate, potassium carbonate, and 18-crown-6 were added. The reaction mixture was boiled under reflux condition. After completion, the reaction mixture was filtered and the reaction mixture was concentrated under reduced pressure, diluted with DCM and washed with saturated $NaHCO_3$ solution and brine. The organic layer was concentrated under reduced pressure and the crude product was purified by automated column chromatography using ethyl acetate/methanol as eluent, followed by prep-SEC purification. **3mPEG15**-Me. A viscous liquid; ^{1}H NMR (500MHz, $CDCl_3$): δ 7.28-7.26 (m, 2H, $(OCH_2CH_2O)_3$-C_6**H_2**-COO), 4.18 (m, 6H, $(OCH_2C$**H_2**$O)_3$-C_6H_2-COO), 3.85 (m, 6H, (OC**H_2**$CH_2O)_3$-C_6H_2-COO), 3.77 (m, 3H, $(OCH_2CH_2O)_3$-C_6H_2-COOC**H_3**), 3.63 (m, 168H, O—C**H_2**C**H_2**-O), 3.36 (m, 9H, O-C**H_3**) ppm; M_n and $Đ$ (GPC): 2702 Da and 1.02; MS (MALDI-TOF): molecular m/z calcd for $C_{101}H_{194}O_{50}$

[**M**]: 2207.26; $C_{101}H_{194}O_{50}$+Na^+ [**M**+Na]$^+$: 2230.25; found 2227.65. Yield: 3.47g, 49%

3mPEG15-COOH: **3mPEG15**-Me was dissolved in THF (0.1 M) and cooled to 0 °C in ice bath. To the solution, 12% aqueous sodium hydroxide solution (0.3 M) was added. The reaction mixture was stirred overnight at room temperature. After completion, 1M HCl was added and the reaction mixture was concentrated under reduced pressure, diluted with DCM and washed with water and brine. The organic layer was dried with Na_2SO_4, filtered, and concentrated under reduced pressure.

3mPEG15-COOH. A viscous solid; ^{1}H NMR (500MHz, $CDCl_3$): δ 7.23 (s, 2H, $(OCH_2CH_2O)_3$-C_6**H_2**-COO), 4.11 (m, 6H, $(OCH_2C$**H_2**$O)_3$-C_6H_2-COO), 3.82-3.32 (m, 174H, (OC**H_2**$CH_2O)_3$-C_6H_2-COO and O—C**H_2**C**H_2**-O), 3.28 (d, 9H, O-C**H_3**) ppm; M_n and $Đ$ (GPC): 2632 Da and 1.02; MS (MALDI-TOF): molecular m/z calcd for $C_{100}H_{192}O_{50}$ [**M**]: 2193.25; $C_{100}H_{192}O_{50}$+Na^+ [**M**+Na]$^+$: 2216.24; found 2213.97. Yield: 3.38g, 98%

Table S2. Molecular weight analysis of functionalized **mPEG15**s and **3mPEG**s.

entry	MALDI-TOF		GPC		
	calculated ([**M**+Na]$^+$)	found ([**M**+Na]$^+$)	M_n^{a} (kDa)	M_w^{a} (kDa)	PDI^a
**mPEG15-OH**	715.41	713.34	0.86	0.88	1.02
**mPEG15-Ts**	869.42	867.23	1.01	1.03	1.02
**3mPEG15-Me**	2230.25	2227.65	2.70	2.75	1.02
**3mPEG15-COOH**	2216.24	2213.97	2.63	2.68	1.02

^{a} GPC was calibrated with a linear PS standard kit

General procedure for the synthesis of **3mPEG15**-*b*-**MAn**

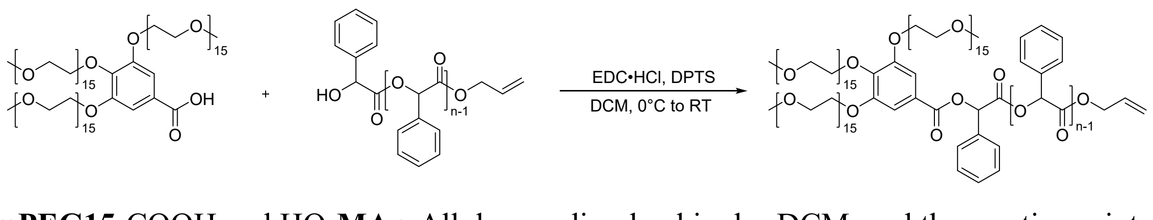

3mPEG15-COOH and HO-**MAn**-Allyl were dissolved in dry DCM, and the reaction mixture was cooled to 0 °C in ice bath. To the solution, 4-(dimethylamino)pyridinium 4-toluenesulfonate (DPTS, 0.3 eq.) and 1-(3-dimethylaminopropyl)-3-ethylcarbodiimide hydrochloride (EDC•HCl, 2.5 eq.) were added, and the reaction mixture was stirred overnight at room temperature. After completion, the reaction mixture was washed with water and brine. The organic layer was dried with Na_2SO_4, filtered, and concentrated under reduced pressure.

The crude product was purified by automated column chromatography using ethyl acetate/methanol as eluent. HO-**MAn**-Allyl was prepared by the desilylation of **MAn** according to the described general procedure.

3mPEG15-*b*-MA64. A white solid; ^{1}H NMR (500MHz, $CDCl_3$): δ 7.44-6.90 (m, 322H, $(OCH_2CH_2O)_3$-C_6**H_2**-COO and Ph-**H**), 6.18-5.85 (m, 64H, COO-C**H**(Ph)C=O), 5.73 (m, 1H, COO-CH_2C**H**CH_2), 5.09 (m, 2H, COO-CH_2CHC**H_2**), 4.53 (m, 2H, COO-C**H_2**$CHCH_2$), 4.17 (m, 6H, $(OCH_2$C**H_2**$O)_3$-C_6H_2-COO), 3.82-3.32 (m, 174H, (OC**H_2**$CH_2O)_3$-C_6H_2-COO and O-C**H_2**C**H_2**-O), 3.38 (d, 9H, O-C**H_3**) ppm; M_n and $Đ$ (GPC): 10540 Da and 1.03; MS (MALDI-TOF): m/z calcd for $C_{615}H_{580}O_{178}$ [**M**]: 10811.63; $C_{615}H_{580}O_{178}$+Na^+ [**M**+Na]$^+$: 10834.62; found 10835.29. Yield: 0.14g, 86%

3mPEG15-*b*-MA96. A white solid; ^{1}H NMR (500MHz, $CDCl_3$): δ 7.43-6.92 (m, 482H, $(OCH_2CH_2O)_3$-C_6**H_2**-COO and Ph-**H**), 6.00 (m, 96H, COO-C**H**(Ph)C=O), 5.73 (m, 1H, COO-CH_2C**H**CH_2), 5.10 (m, 2H, COO-CH_2CHC**H_2**), 4.53 (m, 2H, COO-C**H_2**$CHCH_2$), 4.17 (d, 6H, $(OCH_2$C**H_2**$O)_3$-C_6H_2-COO), 3.64 (m, 174H, (OC**H_2**$CH_2O)_3$-C_6H_2-COO and O-C**H_2**C**H_2**-O), 3.38 (s, 9H, O-C**H_3**) ppm; M_n and $Đ$ (GPC): 14006 Da and 1.03; MS (MALDI-TOF): m/z calcd for $C_{871}H_{772}O_{242}$ [**M**]: 15100.81; $C_{871}H_{772}O_{242}$+Na^+ [**M**+Na]$^+$: 15123.80; found 15125.33. Yield: 0.10g, 71%

3mPEG15-*b*-MA104. A white solid; ^{1}H NMR (500MHz, $CDCl_3$): δ 7.43-6.91 (m, 522H, $(OCH_2CH_2O)_3$-C_6**H_2**-COO and Ph-**H**), 6.00 (m, 104H, COO-C**H**(Ph)C=O), 5.73 (m, 1H, COO-CH_2C**H**CH_2), 5.09 (m, 2H, COO-CH_2CHC**H_2**), 4.48 (m, 2H, COO-C**H_2**$CHCH_2$), 4.17 (d, 6H, $(OCH_2$C**H_2**$O)_3$-C_6H_2-COO), 3.64 (m, 174H, (OC**H_2**$CH_2O)_3$-C_6H_2-COO and O-C**H_2**C**H_2**-O), 3.38 (s, 9H, O-C**H_3**) ppm; M_n and $Đ$ (GPC): 15375 Da and 1.04; MS (MALDI-TOF): m/z calcd for $C_{935}H_{820}O_{258}$ [**M**]: 16173.10; $C_{935}H_{820}O_{258}$+Na^+ [**M**+Na]$^+$: 16196.09; found 16197.48. Yield: 0.02g, 58%

3mPEG15-*b*-MA112. A white solid; ^{1}H NMR (500MHz, $CDCl_3$): δ 7.43-6.91 (m, 562H, $(OCH_2CH_2O)_3$-C_6**H_2**-COO and Ph-**H**), 6.00 (m, 112H, COO-C**H**(Ph)C=O), 5.73 (m, 1H, COO-CH_2C**H**CH_2), 5.09 (m, 2H, COO-CH_2CHC**H_2**), 4.48 (m, 2H, COO-C**H_2**$CHCH_2$), 4.17 (d, 6H, $(OCH_2$C**H_2**$O)_3$-C_6H_2-COO), 3.64 (m, 174H, (OC**H_2**$CH_2O)_3$-C_6H_2-COO and O-C**H_2**C**H_2**-O), 3.38 (s, 9H, O-C**H_3**) ppm; M_n and $Đ$ (GPC): 15526 Da and 1.04; MS (MALDI-TOF): m/z calcd for $C_{999}H_{868}O_{274}$ [**M**]: 17245.40; $C_{999}H_{868}O_{274}$+Na^+ [**M**+Na]$^+$: 17268.39; found 17267.08. Yield: 0.10g, 88%

3mPEG15-*b*-MA128. A white solid; ^{1}H NMR (500MHz, $CDCl_3$): δ 7.43-6.95 (m, 642H,

($OCH_2CH_2O)_3$-C_6**H_2**-COO and Ph-**H**), 6.15-5.86 (m, 128H, COO-C**H**(Ph)C=O), 5.73 (m, 1H, COO-CH_2C**H**CH_2), 5.09 (m, 2H, COO-CH_2CHC**H_2**), 4.53 (m, 2H, COO-C**H_2**$CHCH_2$), 4.17 (d, 6H, (OCH_2C**H_2**$O)_3$-C_6H_2-COO), 3.64 (m, 174H, (OC**H_2**$CH_2O)_3$-C_6H_2-COO and O-C**H_2**C**H_2**-O), 3.38 (s, 9H, O-C**H_3**) ppm; M_n and *Đ* (GPC): 17584 Da and 1.03; MS (MALDI-TOF): m/z calcd for $C_{1127}H_{964}O_{306}$ [**M**]: 19389.99; $C_{1127}H_{964}O_{306}$+Na^+ [**M**+Na]$^+$: 19412.98; found 19409.16. Yield: 0.07g, 65%

3mPEG15-*b*-MA144. A white solid; ^{1}H NMR (500MHz, $CDCl_3$): δ 7.43-6.95 (m, 722H, ($OCH_2CH_2O)_3$-C_6**H_2**-COO and Ph-**H**), 6.15-5.86 (m, 144H, COO-C**H**(Ph)C=O), 5.73 (m, 1H, COO-CH_2C**H**CH_2), 5.09 (m, 2H, COO-CH_2CHC**H_2**), 4.53 (m, 2H, COO-C**H_2**$CHCH_2$), 4.17 (d, 6H, (OCH_2C**H_2**$O)_3$-C_6H_2-COO), 3.64 (m, 174H, (OC**H_2**$CH_2O)_3$-C_6H_2-COO and O-C**H_2**C**H_2**-O), 3.38 (s, 9H, O-C**H_3**) ppm; M_n and *Đ* (GPC): 18042 Da and 1.07; MS (MALDI-TOF): m/z calcd for $C_{1255}H_{1060}O_{338}$ [**M**]: 21534.58; $C_{1255}H_{1060}O_{338}$+Na^+ [**M**+Na]$^+$: 21557.57; found 21584.07. Yield: 0.03g, 50%

3mPEG15-*b*-LMA32. A white solid; ^{1}H NMR (500MHz, $CDCl_3$): δ 7.44-6.90 (m, 162H, ($OCH_2CH_2O)_3$-C_6**H_2**-COO and Ph-**H**), 6.18-5.85 (m, 32H, COO-C**H**(Ph)C=O), 5.73 (m, 1H, COO-CH_2C**H**CH_2), 5.09 (m, 2H, COO-CH_2CHC**H_2**), 4.53 (m, 2H, COO-C**H_2**$CHCH_2$), 4.17 (m, 6H, (OCH_2C**H_2**$O)_3$-C_6H_2-COO), 3.82-3.32 (m, 174H, (OC**H_2**$CH_2O)_3$-C_6H_2-COO and O-C**H_2**C**H_2**-O), 3.38 (d, 9H, O-C**H_3**) ppm; M_n and *Đ* (GPC): 7146 Da and 1.03; MS (MALDI-TOF): m/z calcd for $C_{359}H_{388}O_{114}$ [**M**]: 6522.46; $C_{359}H_{388}O_{114}$+Na^+ [**M**+Na]$^+$: 6545.45; found 6526.73. Yield: 0.95g, 92%

entry	MALDI-TOF		GPC		
	calculated ([**M**+Na]$^+$)	found ([**M**+Na]$^+$)	M_n[a] (kDa)	M_w[a] (kDa)	PDI[a]
**3mPEG15-*b*-MA64**	10834.62	10835.29	10.54	10.86	1.03
**3mPEG15-*b*-MA96**	15123.80	15125.33	14.01	14.43	1.03
**3mPEG15-*b*-MA104**	16196.09	16197.48	15.38	16.00	1.04
**3mPEG15-*b*-MA112**	17268.39	17267.08	15.53	16.15	1.04
**3mPEG15-*b*-MA128**	19412.98	19409.16	17.58	18.11	1.03
**3mPEG15-*b*-MA144**	21557.57	21584.07	18.04	19.30	1.07
**3mPEG15-*b*-LMA32**	6545.45	6526.73	7.15	7.36	1.03

Table S3. Molecular weight analysis of **3mPEG15-*b*-uPMA**s.

[a] GPC was calibrated with a linear PS standard kit

4. Prep-SEC Separation of uPMAs, 3mPEG15-Me, and 3mPEG-*b*-uPMAs

Preparative-Size Exclusion Chromatography (prep-SEC) of uPMAs, 3mPEG15-Me, and 3mPEG-*b*-uPMAs were performed to separate **MAn** and their block copolymers respectively, and unreacted coupling precursors. Prep-SEC was conducted by injecting 5 mL of a polymer solution in $CHCl_3$ (90~100 mg mL^{-1}) to a Recycling Preparative HPLC (LC-9260 NEXT, Japan Analytical Industry) system equipped with JAIGEL-3HR/2.5HR/2HR columns and a differential refractometer. Based on the molecular weight of product, two columns with a series of molecular weight resolutions were used for each purification. Chloroform was used as an eluent with a flow rate of 10.0 mL min^{-1}. Prior to injection, the solution was filtered through a PTFE syringe filter (Whatman, 0.2 μm pore). The SEC was performed under a recycling mode until the coinciding peaks were separated. The desired fraction was collected using a fraction collector. Two prep-SEC systems ran in parallel, giving the maximum capacity of separation of 1 g.

<u>Separation of **MA128**</u>

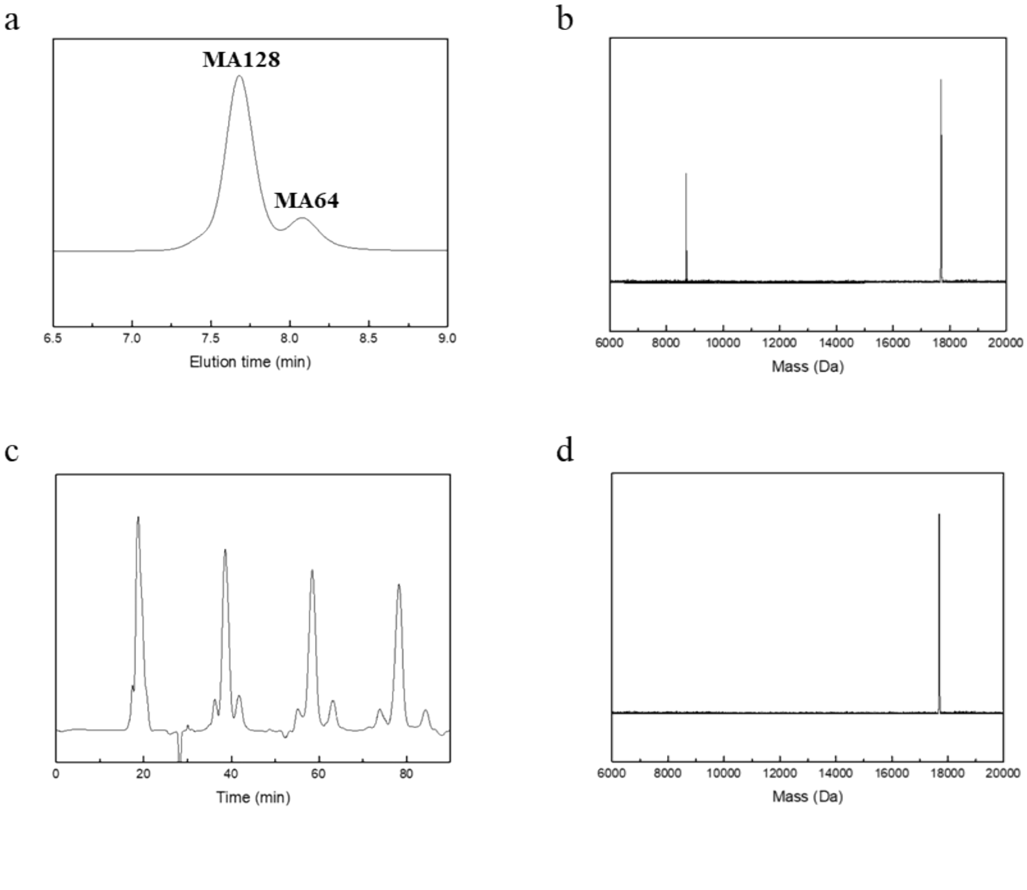

Figure S1. a and b, GPC (a) and MALDI-TOF (b) MS spectra of the crude reaction mixture of **MA128**. **c and d,** prep-SEC traces of the crude mixture during the recycling (c) and MALDI-TOF MS spectrum of **MA128** after prep-SEC (d).

Table S4. Prep-SEC condition for **MA128**

Injection	Column	Flow rate	# of Recycling
300 mg of crude **MA128** in 3 mL of $CHCl_3$	JAIGEL-3HR/2.5HR	10.0 mL/min	3

<u>Separation of **MA112**</u>

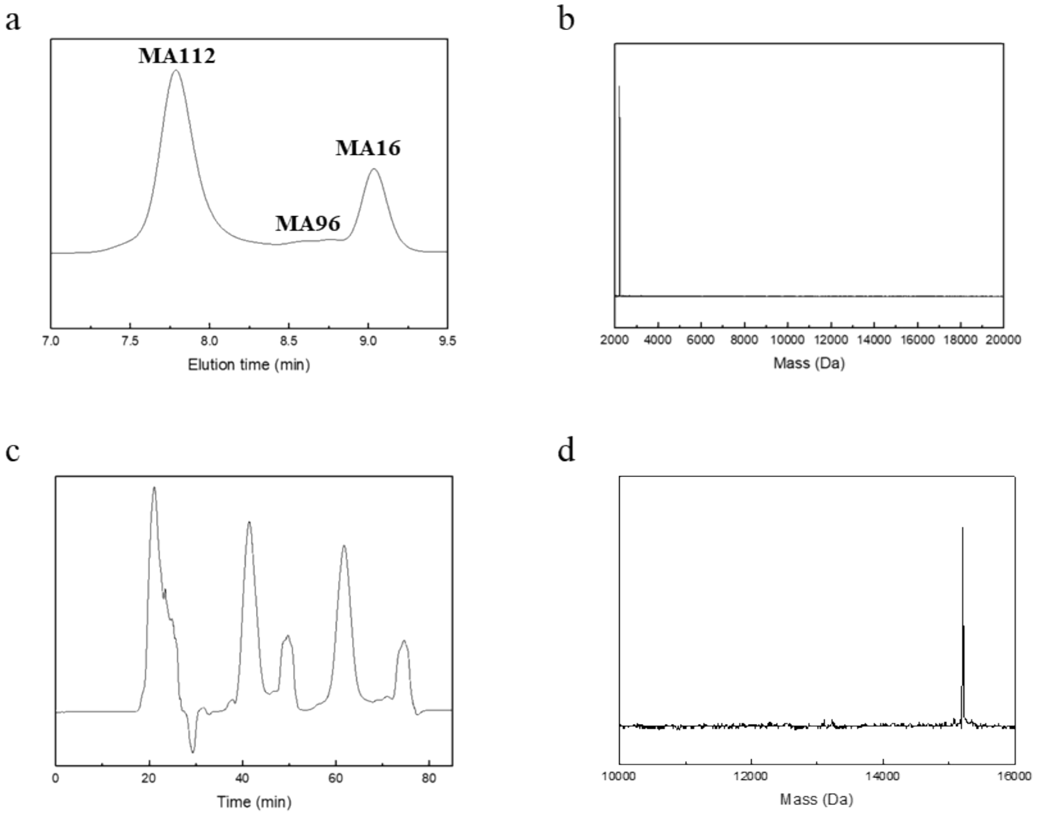

Figure S2. a and b, GPC (a) and MALDI-TOF (b) MS spectra of the crude reaction mixture of **MA112**. **c and d,** prep-SEC traces of the crude mixture during the recycling (c) and MALDI-TOF MS spectrum of **MA128** after prep-SEC (d).

Table S5. Prep-SEC condition for **MA112**

Injection	Column	Flow rate	# of Recycling
200 mg of crude **MA112** in 2 mL of $CHCl_3$	JAIGEL-3HR/2.5HR	10.0 mL/min	2

<u>Separation of **3mPEG15**-Me</u>

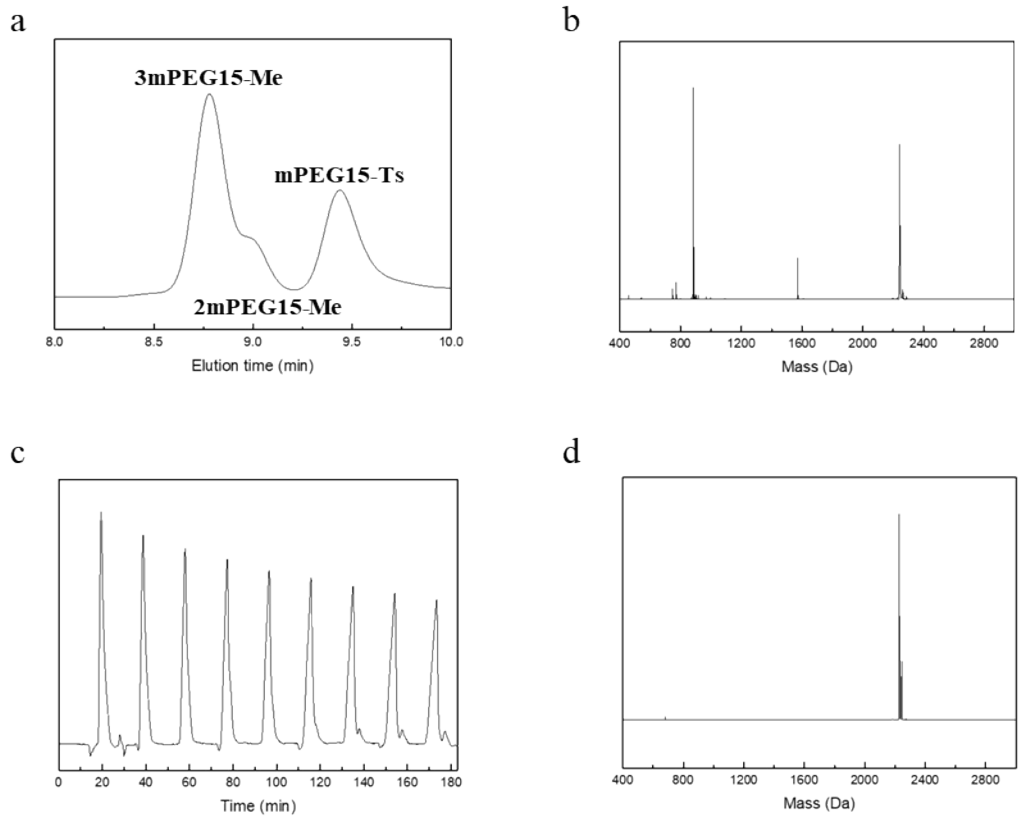

Figure S3. a and b, GPC (a) and MALDI-TOF (b) MS spectra of the crude reaction mixture of **3mPEG15-**Me. **c and d,** prep-SEC traces of the crude mixture during the recycling (c) and MALDI-TOF MS spectrum of **3mPEG**-Me after prep-SEC (d).

Table S6. Prep-SEC condition for **3mPEG15-**Me

Injection	Column	Flow rate	# of Recycling
200 mg of crude **3mPEG15**-Me in 2 mL of $CHCl_3$	JAIGEL-2.5HR/2HR	10.0 mL/min	8

Separation of **3mPEG15-*b*-MA128**

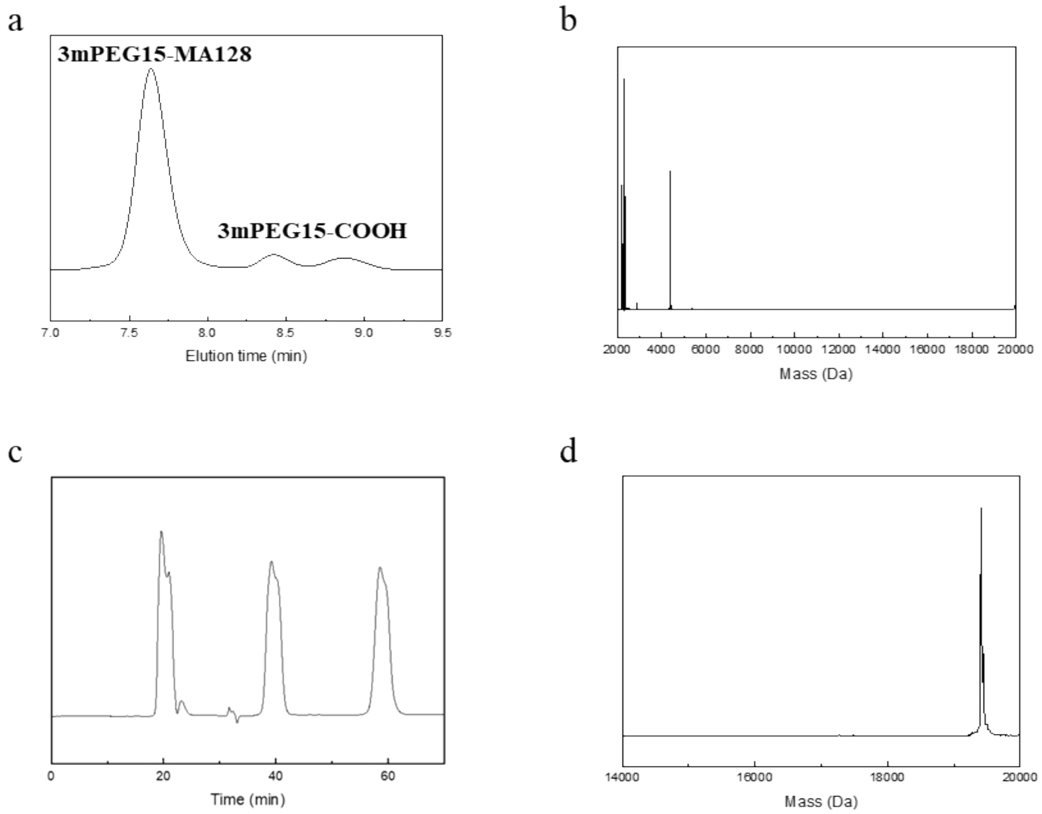

Figure S4. a and b, GPC (a) and MALDI-TOF (b) MS spectra of the crude reaction mixture of **3mPEG15-MA128**. **c and d,** prep-SEC traces of the crude mixture during the recycling (c) and MALDI-TOF MS spectrum of **3mPEG15-MA128** after prep-SEC (d).

Table S7. Prep-SEC condition for **3mPEG15-*b*-MA128**

Injection	Column	Flow rate	# of Recycling
200 mg of crude **3mPEG15-*b*-MA128** in 2 mL of $CHCl_3$	JAIGEL-3HR/2.5HR	10.0 mL/min	2

5. NMR Spectra of uPMAs, mPEG15s, and their block copolymers

^{1}H NMR spectra of **MAn**

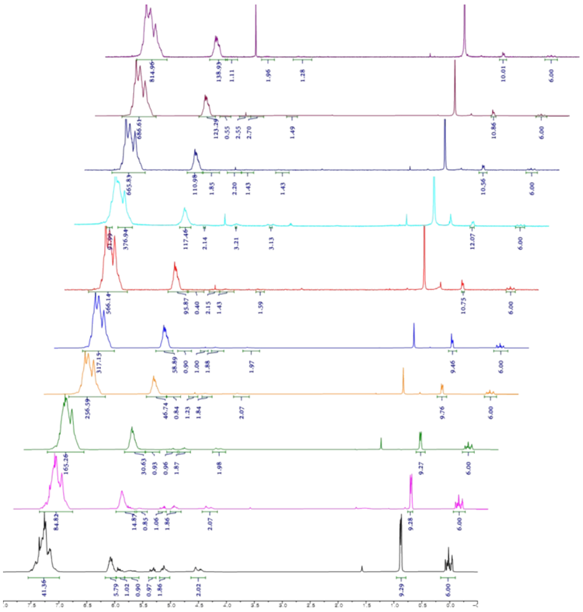

Figure S5. ^{1}H NMR spectra of **MA8** (black), **MA16** (magenta), **MA32** (green), **MA48** (orange), **MA64** (blue), **MA96** (red), **MA104** (cyan), **MA112** (navy), **MA128** (dark purple), and **MA144** (purple).

^{13}C NMR spectra of **MAn**

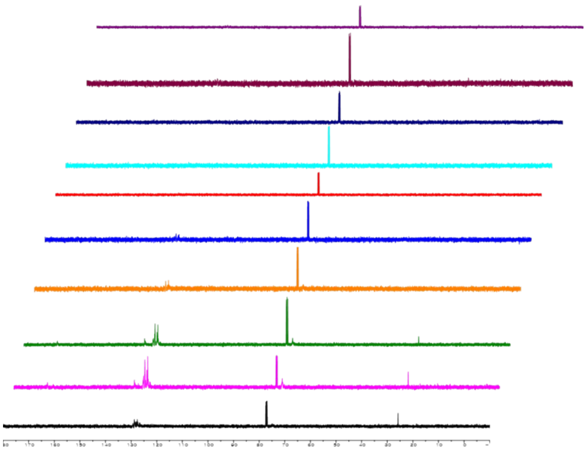

Figure S6. ^{13}C NMR spectra of **MA8** (black), **MA16** (magenta), **MA32** (green), **MA48** (orange), **MA64** (blue), **MA96** (red), **MA104** (cyan), **MA112** (navy), **MA128** (dark purple), and **MA144** (purple).

<u>^{1}H NMR spectra of stereospecific **MAn**</u>

a

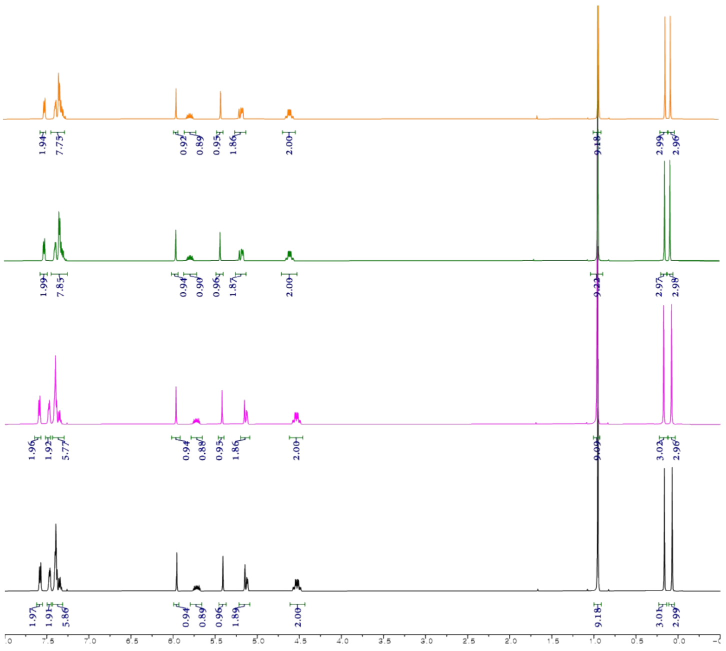

b

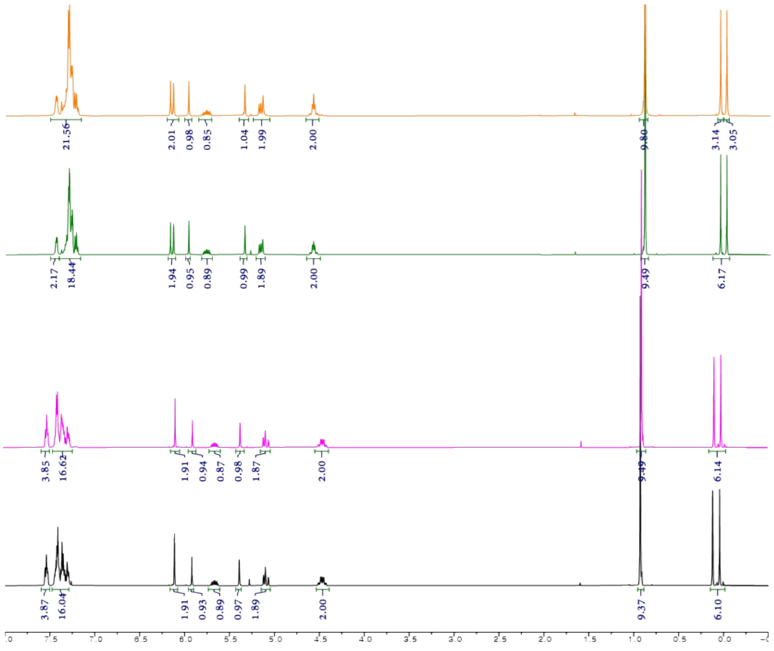

Figure S7. ^{1}H NMR spectra of (a) **LMA2** (black), **DMA2** (magenta), **LDMA2** (green), and **DLMA2** (orange). (b) **LMA4** (black), **DMA4** (magenta), **LDMA4** (green), and **DLMA4** (orange).

<u>^{13}C NMR spectra of stereospecific **MAn**</u>

a

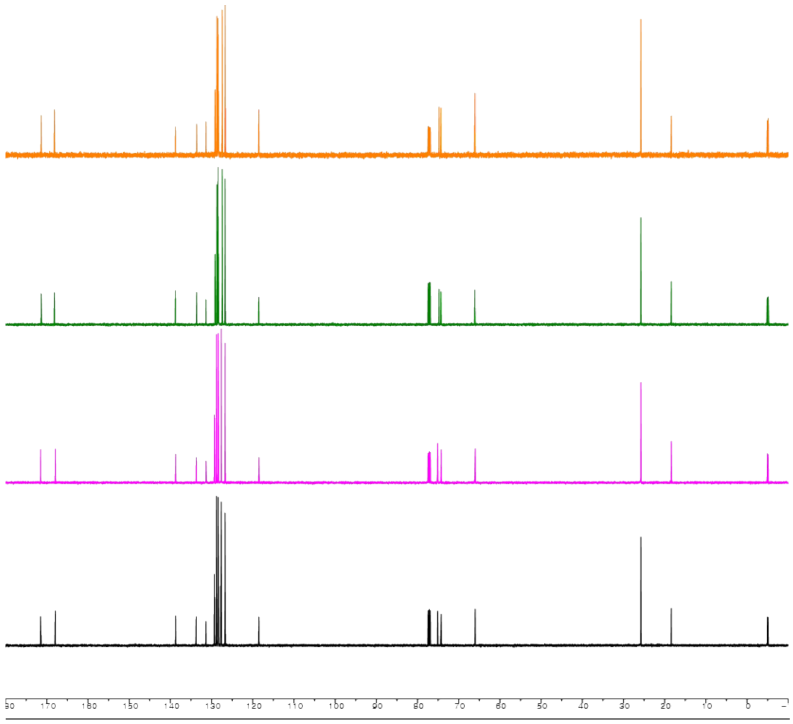

b

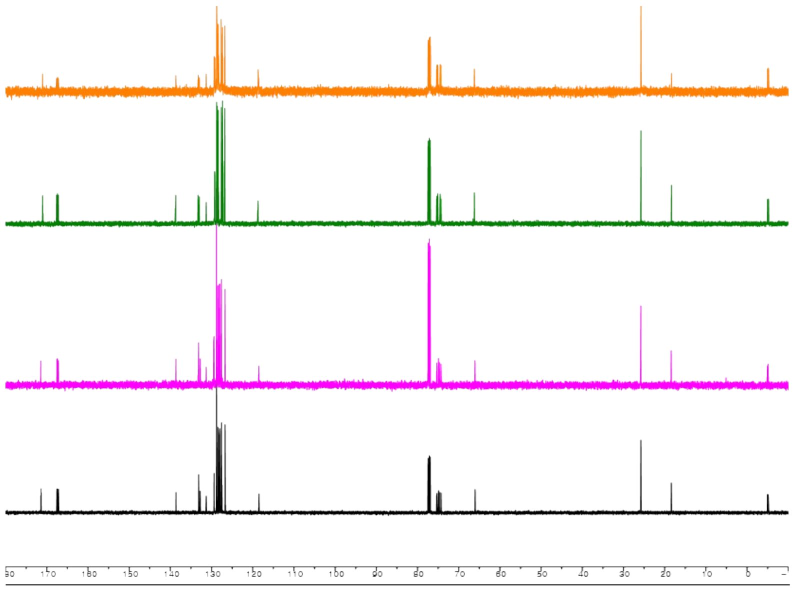

Figure S8. ^{13}C NMR spectra of (a) **LMA2** (black), **DMA2** (magenta), **LDMA2** (green), and **DLMA2** (orange). (b) **LMA4** (black), **DMA4** (magenta), **LDMA4** (green), and **DLMA4** (orange).

<u>^{1}H NMR spectra of **mPEG15s** and **3mPEG15s**</u>

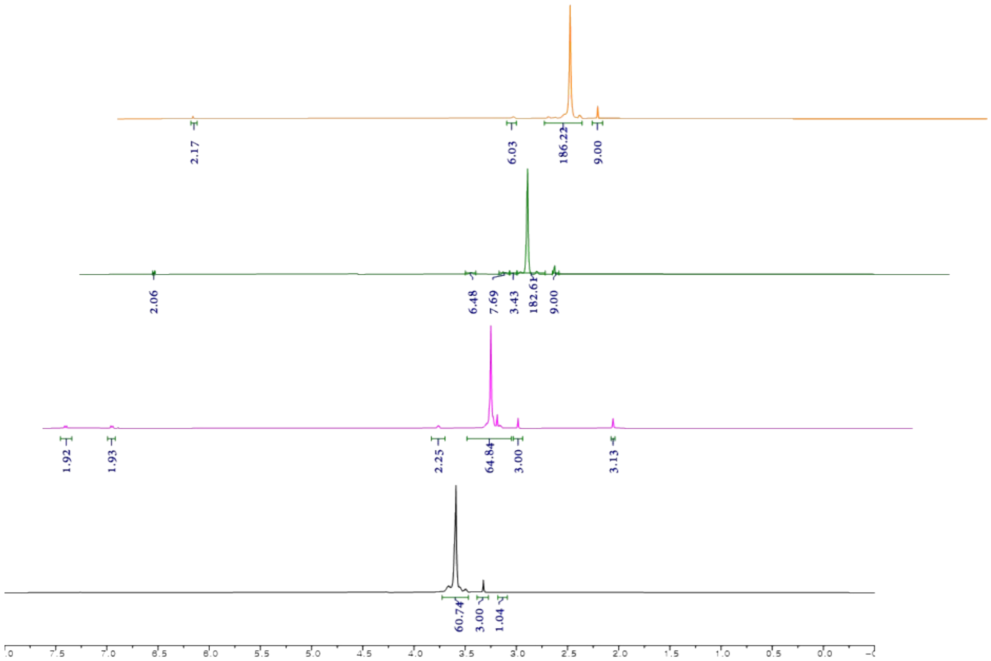

Figure S9. ^{1}H NMR spectra of **mPEG15**-OH (black), **mPEG15**-Ts (magenta), **3mPEG15**-Me (green), and **3mPEG15**-COOH (orange).

<u>^{13}C NMR spectra of **mPEG15s** and **3mPEG15s**</u>

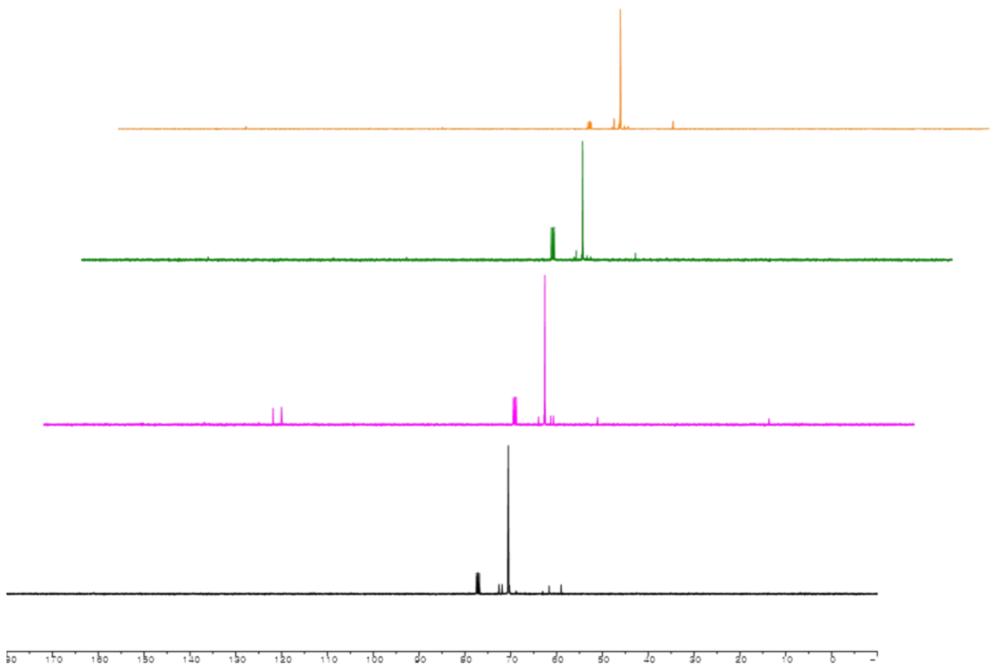

Figure S10. ^{13}C NMR spectra of **mPEG15**-OH (black), **mPEG15**-Ts (magenta), **3mPEG15**-Me (green), and **3mPEG15**-COOH (orange).

<u>^{1}H NMR spectra of **3mPEG15-*b*-MAn**</u>

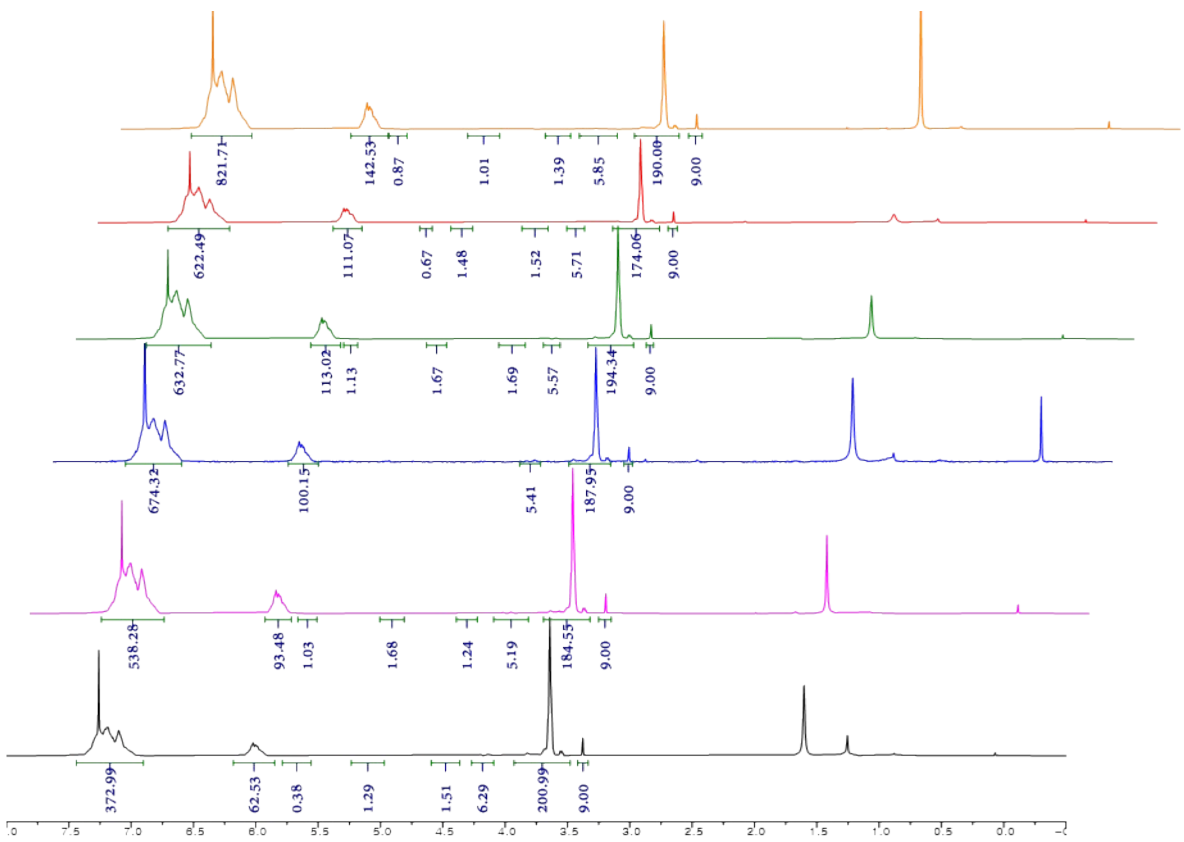

Figure S11. ^{1}H NMR spectra of **3mPEG15-*b*-MA64** (black), **3mPEG15-*b*-MA96** (magenta), **3mPEG15-*b*-MA104** (blue), **3mPEG15-*b*-MA112** (green), **3mPEG15-*b*-MA128** (red), and **3mPEG15-*b*-MA144** (orange).

6. GPC chromatograms of uPMAs and 3mPEGs

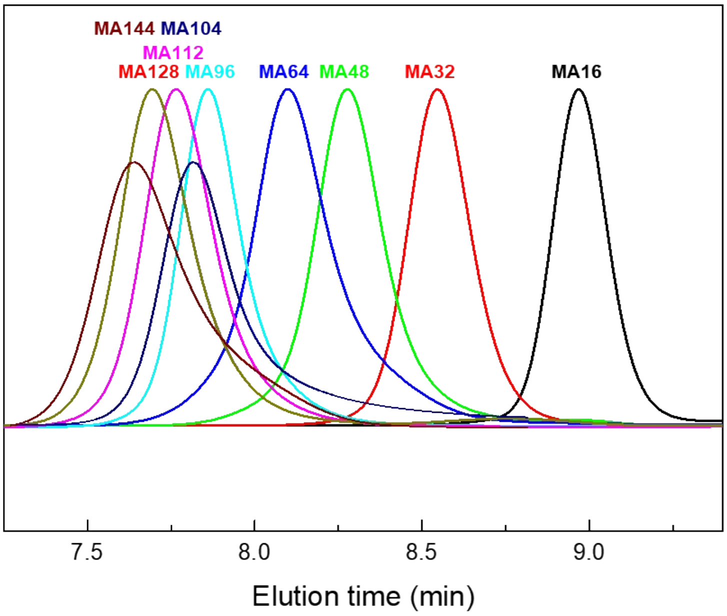

Figure S12. Combined GPC chromatograms of uPMAs.

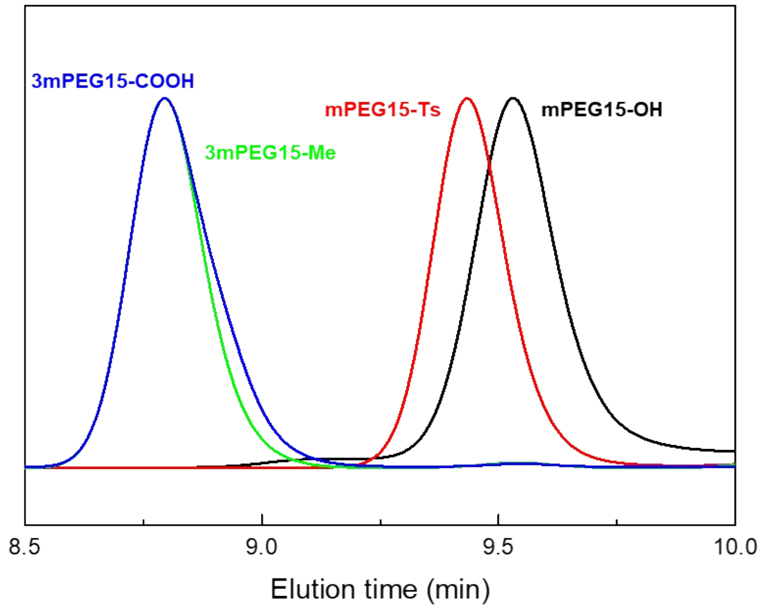

Figure S13. Combined GPC chromatograms of mPEG15s and 3mPEG15s.

7. MALDI-TOF Mass Spectra of uPMAs mPEGs, and their block copolymers

<u>MALDI-TOF Mass spectra of **MAns**</u>

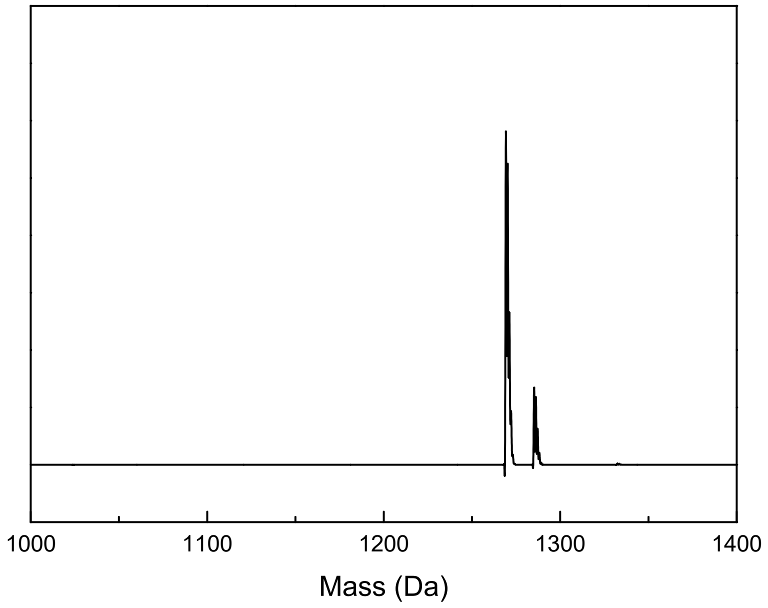

Figure S14. MALDI-TOF mass spectrum of **MA8**. m/z calcd for $C_{73}H_{68}O_{17}Si$ [**M**]: 1244.42; found 1269.27.

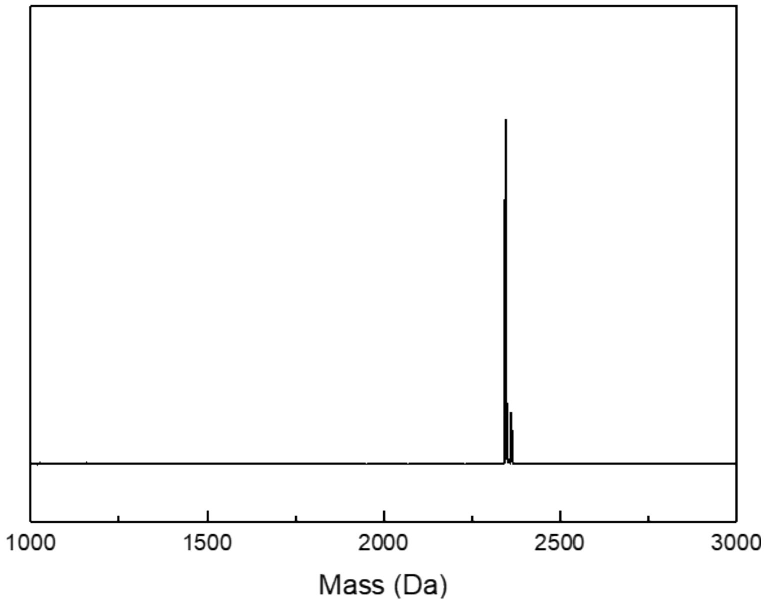

Figure S15. MALDI-TOF mass spectrum of **MA16**. m/z calcd for $C_{137}H_{116}O_{33}Si$ [**M**]: 2316.72; found 2342.80.

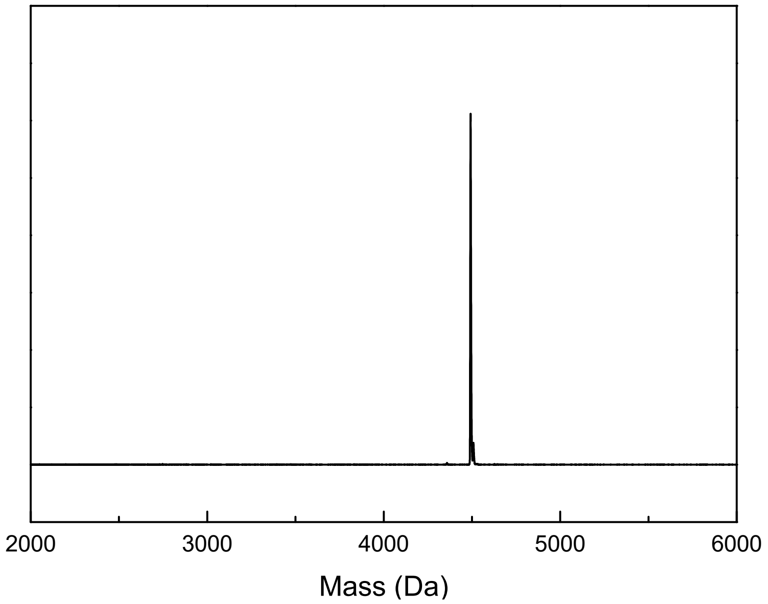

Figure S16. MALDI-TOF mass spectrum of **MA32.** molecular m/z calcd for $C_{265}H_{212}O_{65}Si$ [**M**]: 4461.31; found 4492.33

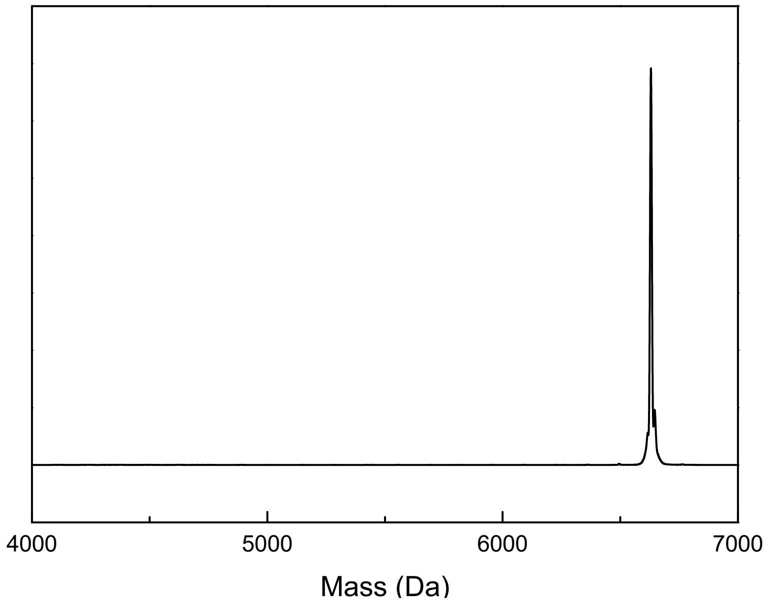

Figure S17. MALDI-TOF mass spectrum of **MA48.** molecular m/z calcd $C_{393}H_{308}O_{97}Si$ [**M**]: 6605.89; found 6630.36.

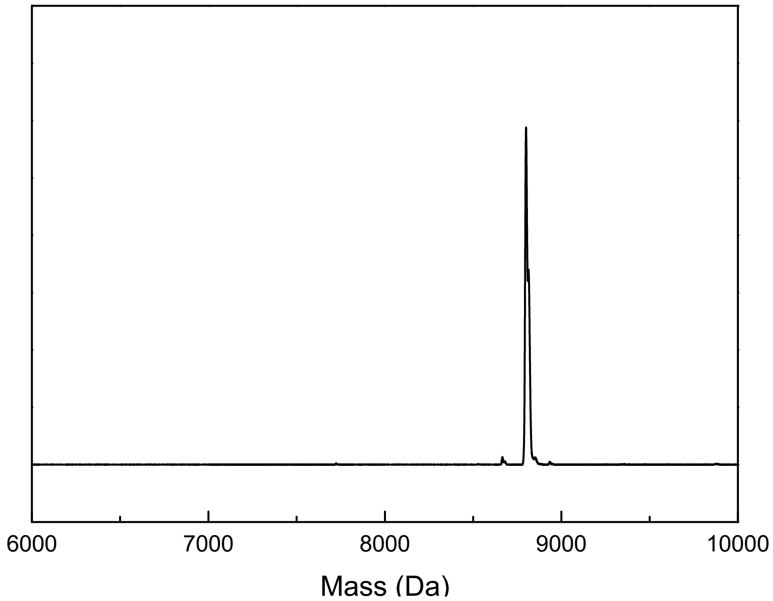

Figure S18. MALDI-TOF mass spectrum of **MA64.** molecular m/z calcd for $C_{521}H_{404}O_{129}Si$ [**M**]: 8750.48; found 8799.84.

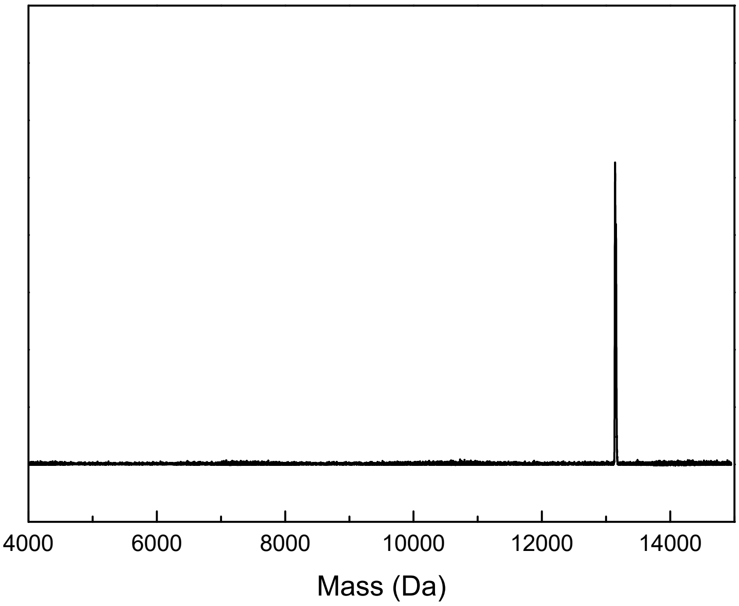

Figure S19. MALDI-TOF mass spectrum of **MA96.** molecular m/z calcd for $C_{777}H_{596}O_{193}Si$ [**M**]: 13039.66; found 13188.54.

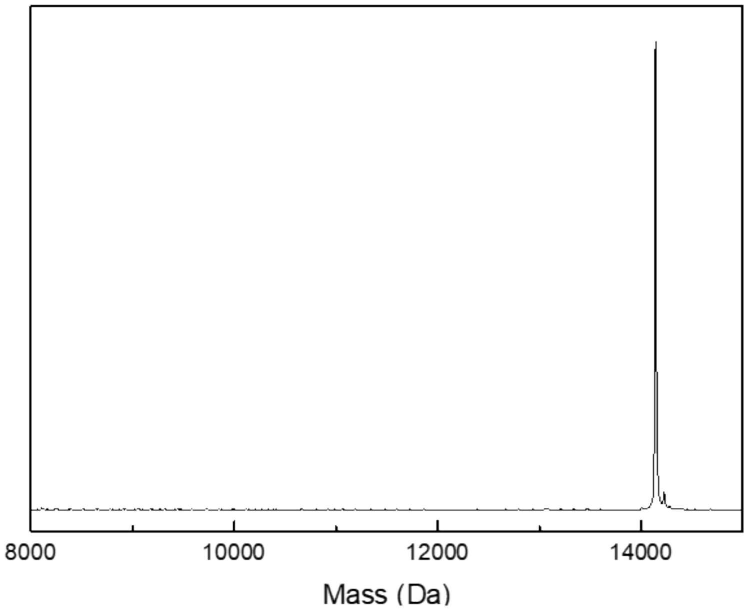

Figure S20. MALDI-TOF mass spectrum of **MA104.** molecular m/z calcd for $C_{841}H_{644}O_{209}Si$ [**M**]: 14111.95; found 14141.88.

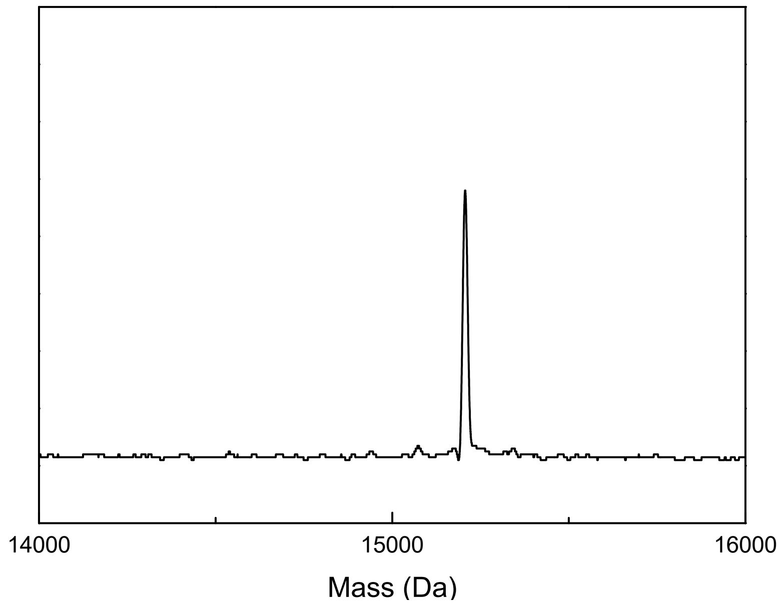

Figure S21. MALDI-TOF mass spectrum of **MA112.** molecular m/z calcd for $C_{905}H_{692}O_{225}Si$ [**M**]: 15184.25; found 15207.02.

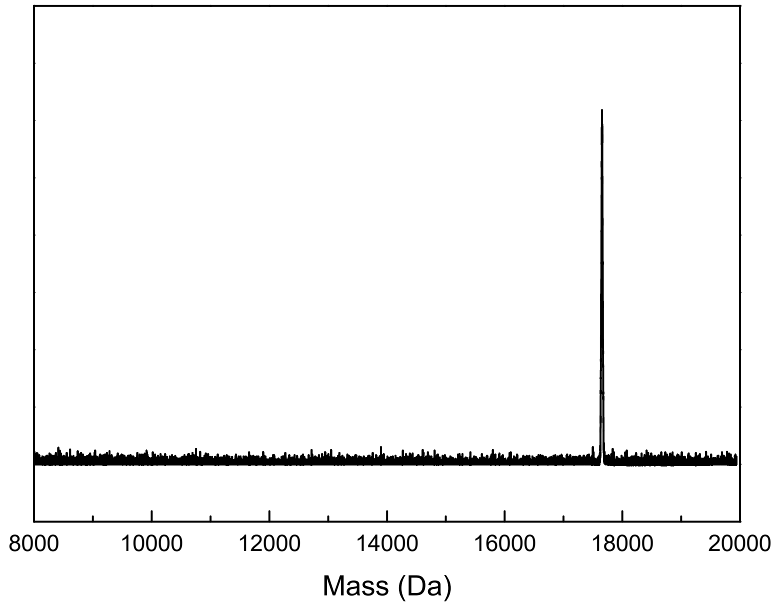

Figure S22. MALDI-TOF mass spectrum of **MA128.** molecular m/z calcd for $C_{1033}H_{788}O_{257}Si$ [**M**]: 17328.84; found 17710.88.

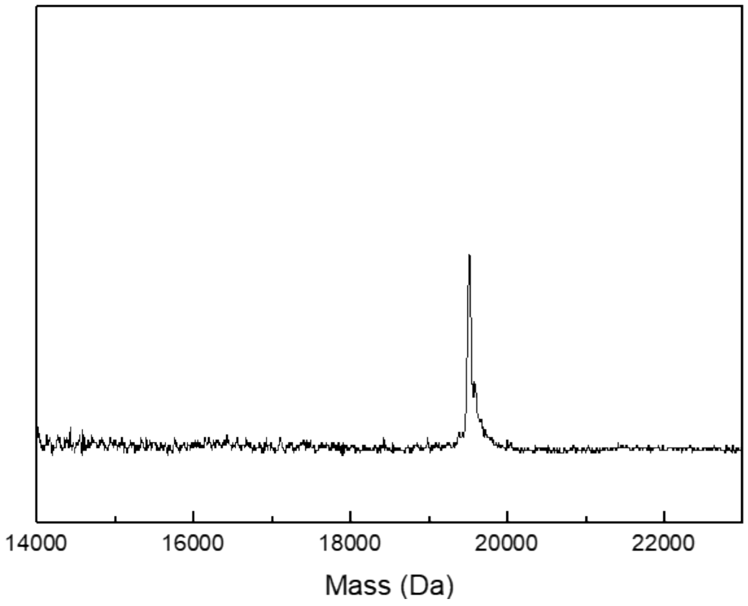

Figure S23. MALDI-TOF mass spectrum of **MA144.** molecular m/z calcd for $C_{1255}H_{1060}O_{338}Si$ [**M**]: 21534.58; found 19515.13.

<u>MALDI-TOF Mass spectra of **mPEG15s and 3mPEG15s**</u>

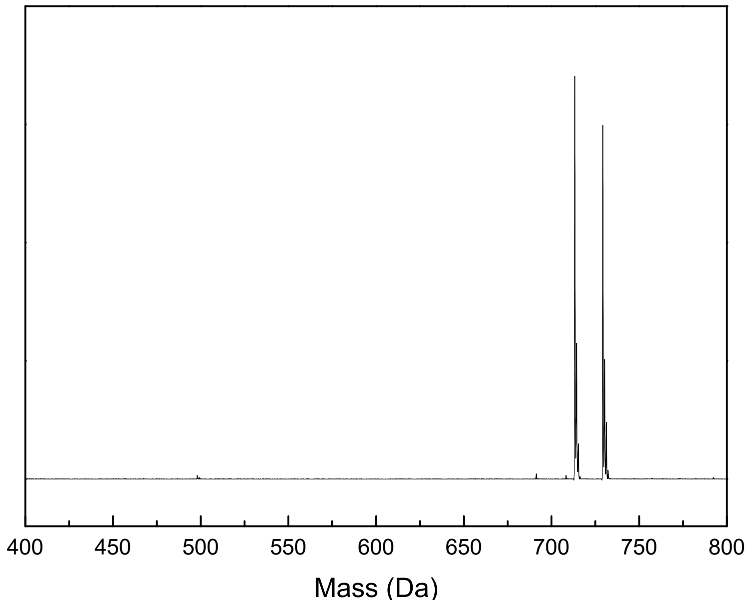

Figure S24. MALDI-TOF mass spectrum of **mPEG15**-OH. m/z calcd for $C_{31}H_{64}O_{16}$ [**M**]: 692.42; found 713.34.

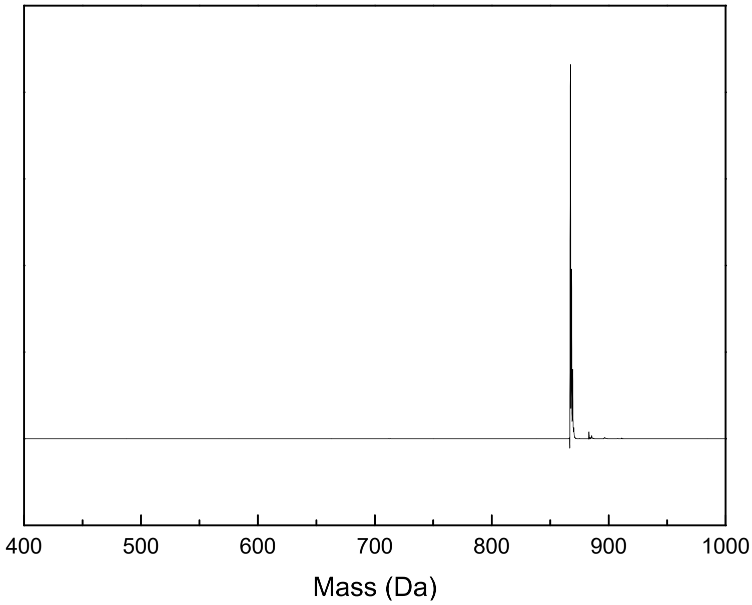

Figure S25. MALDI-TOF mass spectrum of **mPEG15**-Ts. m/z calcd for $C_{38}H_{70}O_{18}S$ [**M**]: 846.43; found 867.23.

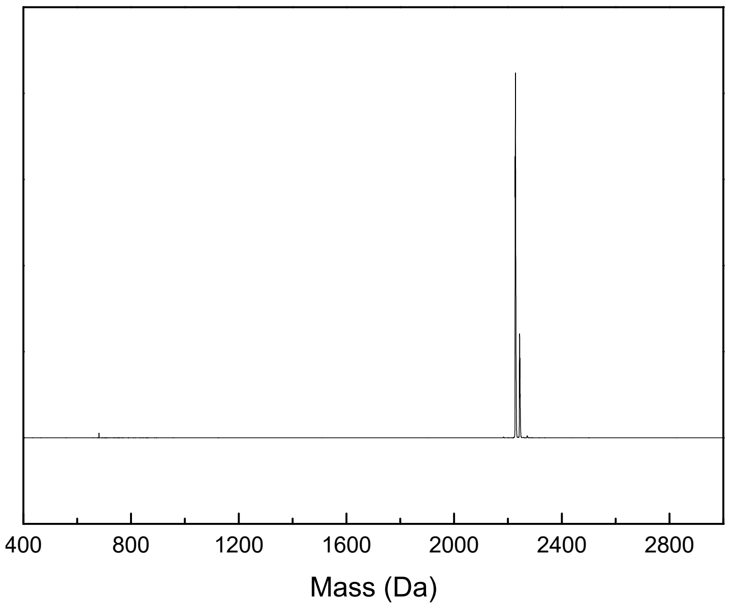

Figure S26. MALDI-TOF mass spectrum of **3mPEG15**-Me. m/z calcd for $C_{101}H_{194}O_{50}$ [**M**]: 2207.26; found 2227.65.

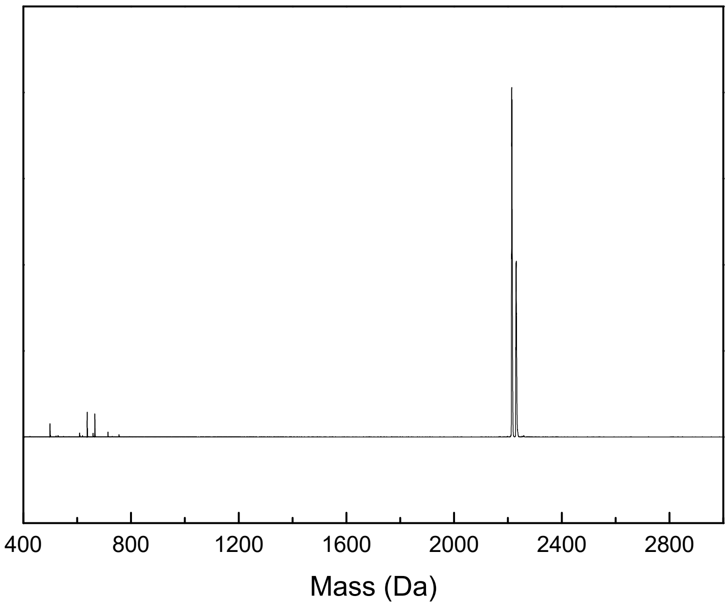

Figure S27. MALDI-TOF mass spectrum of **3mPEG15**-COOH. m/z calcd for $C_{100}H_{192}O_{50}$ [**M**]: 2193.25; found 2213.97.

MALDI-TOF Mass spectra of **3mPEG-*b*-MAns**

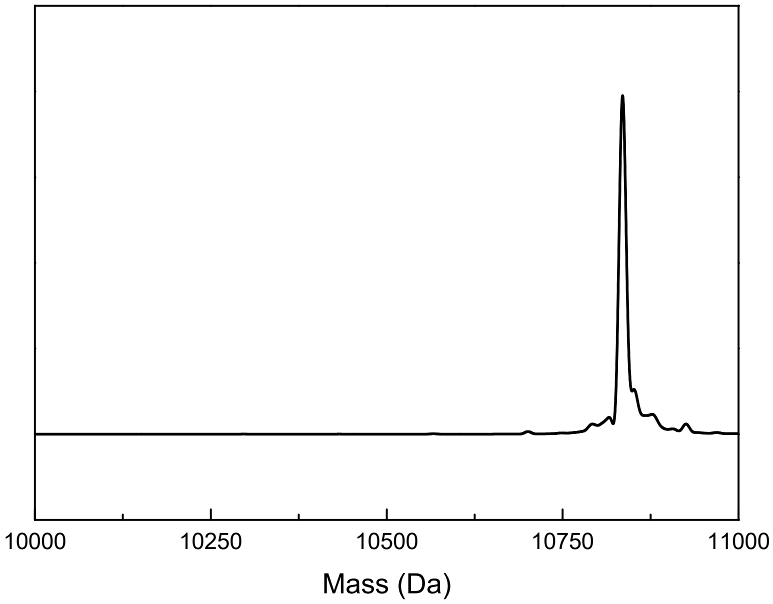

Figure S28. MALDI-TOF mass spectrum of **3mPEG15-*b*-MA64**. m/z calcd for $C_{615}H_{580}O_{178}$ [**M**]: 10811.63; found 10835.29.

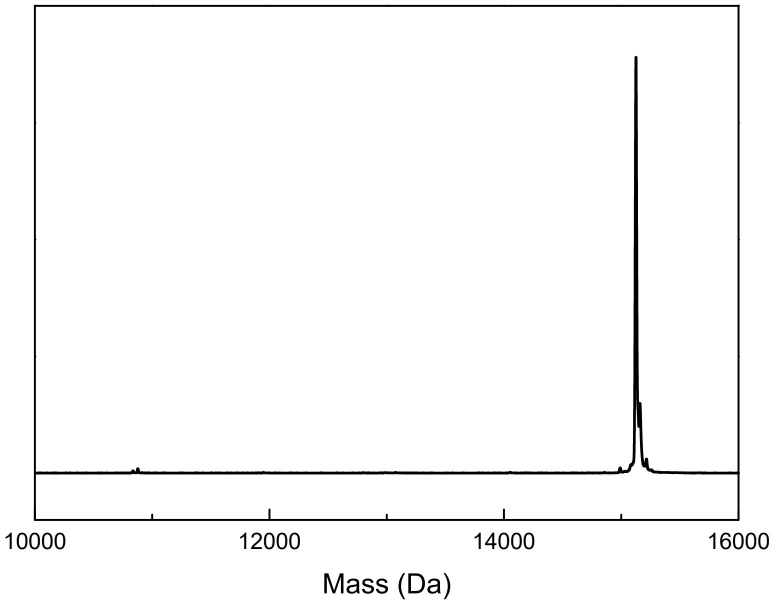

Figure S29. MALDI-TOF mass spectrum of **3mPEG15-*b*-MA96**. m/z calcd for $C_{871}H_{772}O_{242}$ [**M**]: 15100.81; found 15125.33.

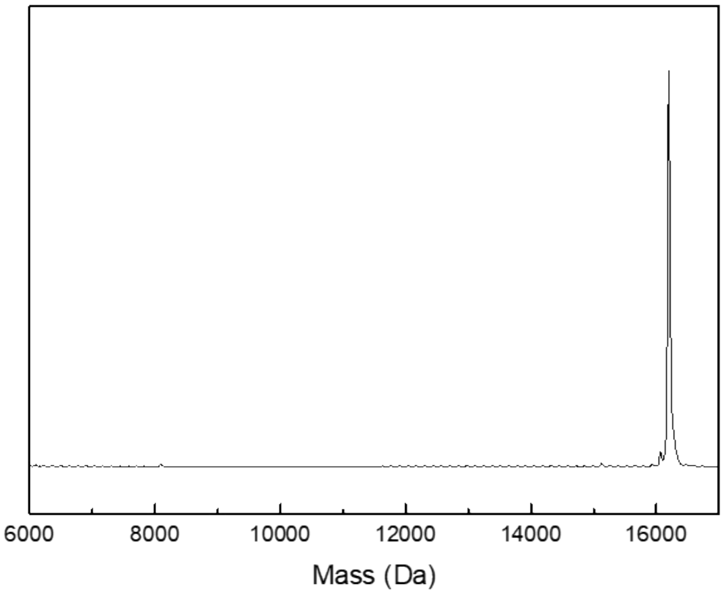

Figure S30. MALDI-TOF mass spectrum of **3mPEG15-*b*-MA104**. m/z calcd for $C_{935}H_{820}O_{258}$ [**M**]: 16173.10; found 16197.48.

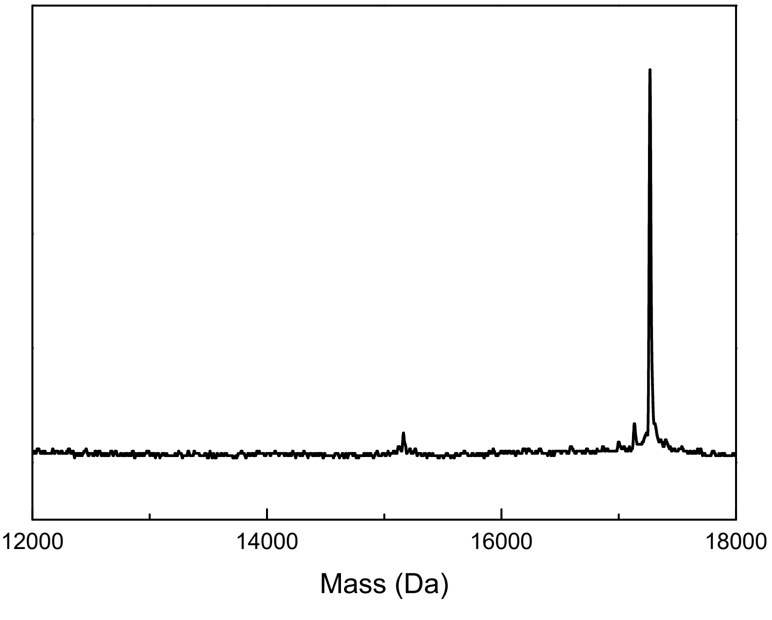

Figure S31. MALDI-TOF mass spectrum of **3mPEG15-*b*-MA112**. m/z calcd for $C_{999}H_{868}O_{274}$ [**M**]: 17245.40; found 17267.08.

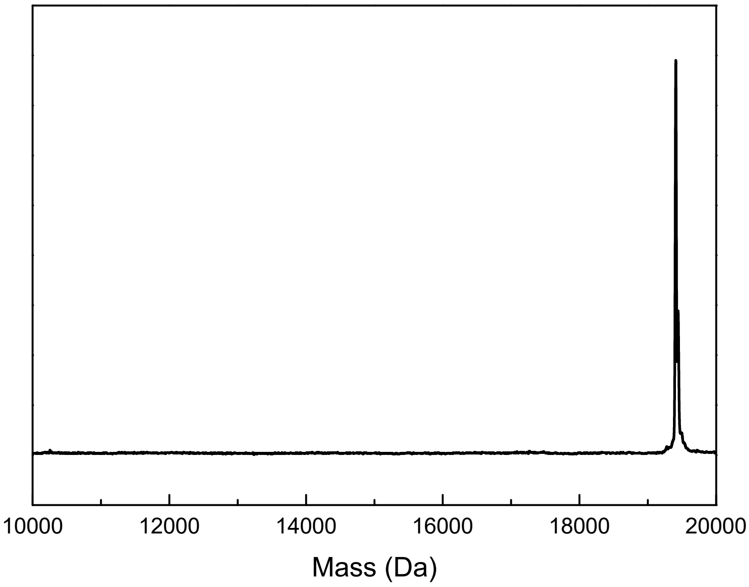

Figure S32. MALDI-TOF mass spectrum of **3mPEG15-*b*-MA128**. m/z calcd for $C_{1127}H_{964}O_{306}$ [**M**]: 19389.99; found 19409.16.

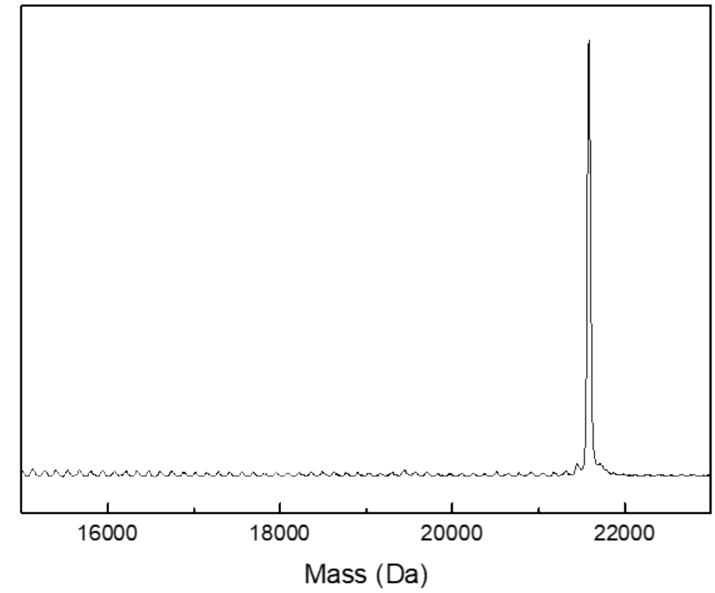

Figure S33. MALDI-TOF mass spectrum of **3mPEG15-*b*-MA144**. m/z calcd for $C_{1255}H_{1060}O_{338}$ [**M**]: 21534.58; found 21584.07.

8. DSC Results of Linear uPMAs

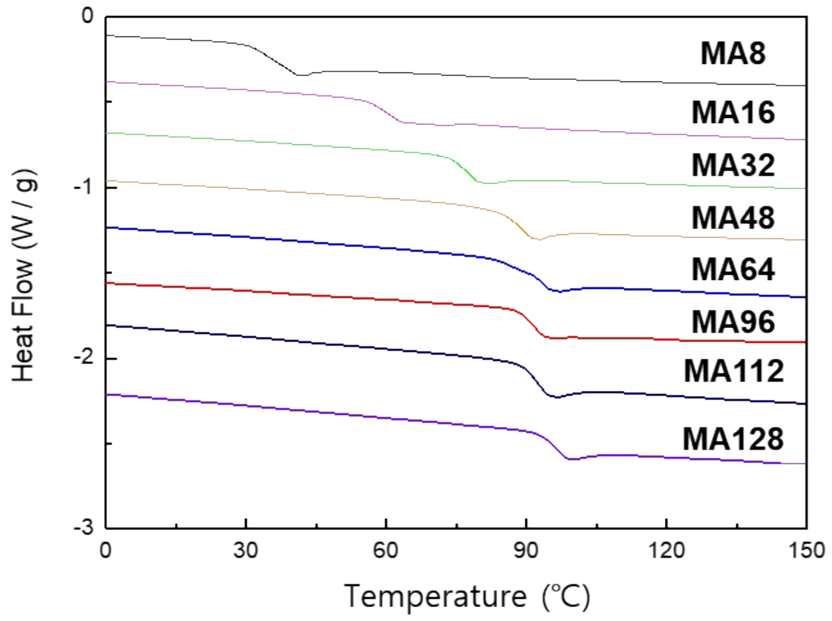

Figure S34. 3rd heating scans of a series of **MAn**.

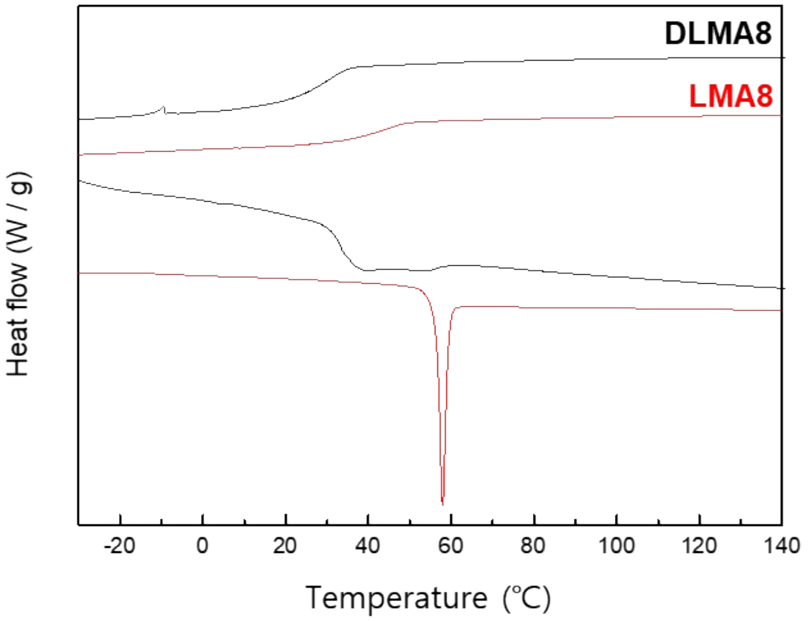

Figure S35. 1st heating/freezing scan of octameric isotactic **LMA8** (red) and syndiotactic **DLMA8** (black).

9. Solution Self-assembly and crystallization-driven self-assembly of Block Copolymers

Solution self-assembly: The target BCP (5 mg) was dissolved in acetone (1 mL) in a 4 mL capped vial with a magnetic stirrer bar. The solution was stirred for 1 h at room temperature (330 rpm). Then DI water in 6mL syringe was directly introduced to acetone solution of BCPs by using syringe pump (1mL/hr). The resulting suspension was subjected to dialysis (molecular weight cutoff 12–14 kDa (SpectraPor, Rancho Dominguez, CA)) against water for 24 h and microstructures were analyzed by TEM and SEM.

Solvent diffusion-evaporation self-assembly (SDESA): The target BCP (0.5 mg) was dissolved in dioxane (1 mL) in a small aluminum basket with solvent level lower. 40 mL vial was saturated by 5 mL of DI water and BCP solution-containing aluminum basket was located inside of the vial for 24 h. After the solution became cloudy, the resulting suspension was subjected to dialysis (molecular weight cutoff 12–14 kDa (SpectraPor, Rancho Dominguez, CA)) against water for 24 h and microstructures were analyzed by TEM and SEM.

Crystallization-driven self-assembly (CDSA) of **3mPEG-*b*-LMA32** was performed.[2] 1 mg of **3mPEG-*b*-LMA32** was added to 1mL of acetonitrile in 4mL vial and sealed. The sample was heated in oil bath at 90 °C, followed by a slow cooling and equilibrium at 25 °C without perturbation for 24h. The resulting solution was diluted by acetonitrile and studied by TEM.

Using Image J program, diameters of more than 100 crystals and particles were measured. The number-average diameter (L_n) and weight-average diameter (L_w) were quantitatively deduced from these data according to the following equation (L = crystal diameter, N = number).[3]

$$L_n = \frac{\sum_{i=1}^{n} N_i L_i}{\sum_{i=1}^{n} N_i} \qquad L_w = \frac{\sum_{i=1}^{n} N_i L_i^2}{\sum_{i=1}^{n} N_i L_i} \qquad \frac{L_w}{L_n} - 1 = \left(\frac{\sigma}{L_n}\right)^2$$

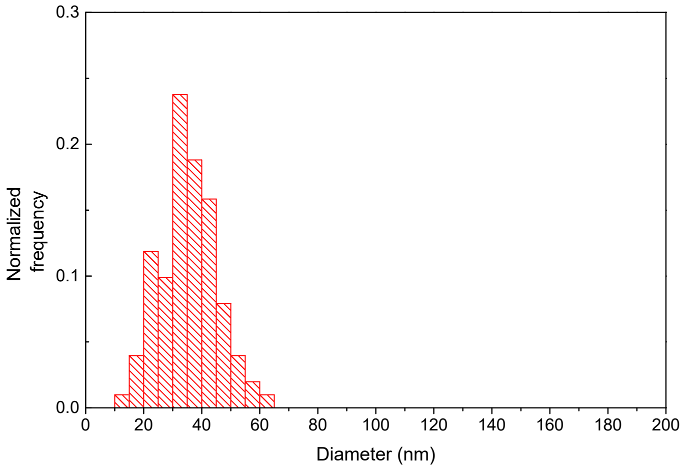

Figure S36. Histogram of the diameter distribution of 3mPEG15-*b*-LMA32 crystalline structures. Average diameter is 34.8 nm and PDI is 1.08.

Table S8. Summary of diameter analysis for 3mPEG15-*b*-PMAs

Entry	L_n (nm)	L_w (nm)	L_w / L_n	σ (nm)
3mPEG15-*b*-LMA32 (CDSA)	34.8	37.5	1.08	9.7
3mPEG15-*b*-MA96 (SDESA)	6566.0	6823.6	1.04	1300.5
3mPEG15-*b*-MA112 (SDESA)	4041.4	4300.9	1.06	1024.2
3mPEG15-*b*-MA128 (SDESA)	2488.2	2642.7	1.06	620.0

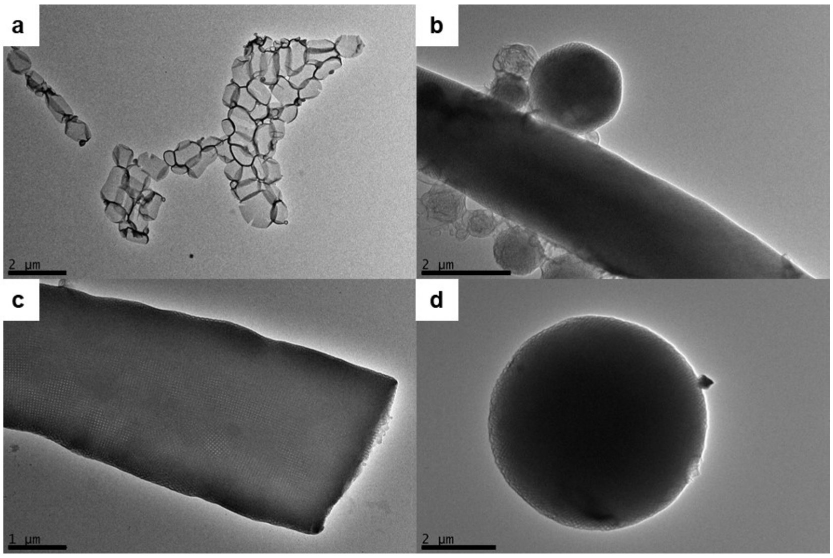

Figure S37. TEM images of solution self-assembled 3mPEG-*b*-uPMAs in acetone with 20% dioxane. (a) Flat bilayers of 3mPEG-*b*-MA104. (b) Polymer cubosomes and cubic phase chunk of 3mPEG-*b*-MA112. (c) Cubic phase chunk of 3mPEG15-*b*-MA128. (d) Polymer cubosomes of 3mPEG15-*b*-MA144.

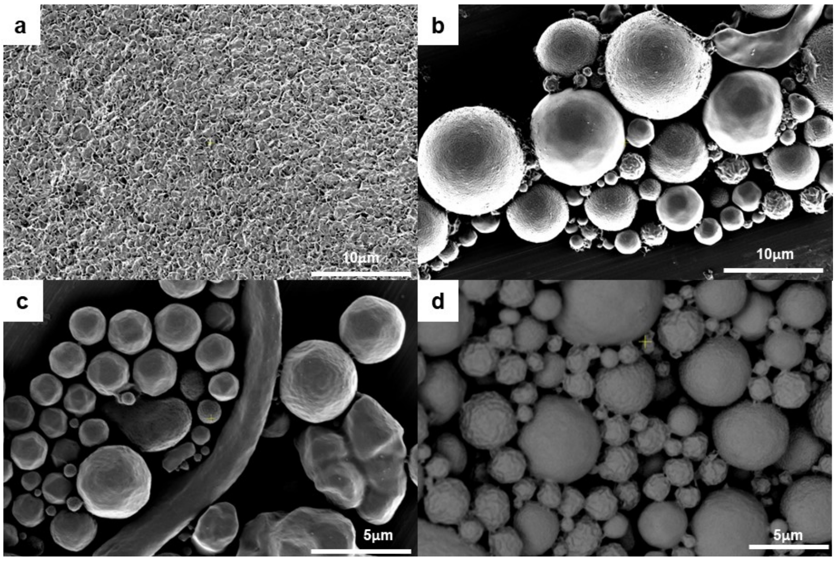

Figure S38. SEM images of solution self-assembled 3mPEG-*b*-uPMAs in acetone with 20% dioxane. (a) Flat bilayers of 3mPEG-*b*-MA104. (b) Polymer cubosomes and cubic phase chunk of 3mPEG-*b*-MA112. (c) Polymer cubosomes and cubic phase chunk of 3mPEG15-*b*-MA128. (d) Polymer cubosomes of 3mPEG15-*b*-MA144.

10. References

(1) A. M. Wawro, T. Muraoka, M. Kato, and K. Kinbara, *Org. Chem. Front.*, 2016, **3**, 1524-1534.

(2) Y. Kwon, and K. T. Kim, *Macromolecules*, 2021, **54**, 10487-10498.

(3) Q. Shi, C. Song, M. Chen, J. Xu, S. Zheng, J. Tan, J. Zhang, N. Wang, J. Hu, and S. Liu, *J. Am. Chem. Soc.*, 2023, **145**, 23176-23187; J. C. Worch, H. Prydderch, S. Jimaja, P. Bexis, M. L. Becker, and A. P. Dove, *Nat. Rev. Chem.*, 2019, **3**, 514-535; D. Bandelli, J. Alex, C. Weber, and U. S. Schubert, *Macromol. Rapid Commun.*, 2020, **41**, 1900560.
